# Proteasomal inhibition compromises microvascular integrity via distinct effects on non-immune endothelial cells and immune cells

**DOI:** 10.3389/fimmu.2026.1759368

**Published:** 2026-05-19

**Authors:** Prajakta Sawant, Hannah Wolf, Aleena Mathew, Johanna Bensalel, Julio Gallego-Delgado, Pratyusha Mandal

**Affiliations:** 1 Doctoral Program in Biology (Molecular, Cellular & Developmental Biology), The Graduate Center, The City University of New York, New York, NY, United States; 2 Department of Biological Sciences, Bronx, Lehman College, The City University of New York, New York, NY, United States; 3 Doctoral Program in Biochemistry, The Graduate Center, The City University of New York, New York, NY, United States

**Keywords:** caspase, endoplasmic reticulum, microvascular endothelial cells, mitochondria, multiple myeloma, PBMC, proteasomal inhibitor, TNF

## Abstract

Chemotherapeutic proteasomal inhibitors Bortezomib (BTZ, reversible inhibitor) and Carfilzomib (CFZ, irreversible inhibitor) are established frontline therapies for multiple myeloma (MM). However, their on-target versus off-target bystander effects in the bloodstream are not clearly known. To elucidate the acute adverse effects of intravenous proteasomal inhibition, we studied some of the key cell types of blood microvasculature: barrier endothelial cells, peripheral blood mononuclear cells (PBMC)s and multiple myeloma (MM1.S) cells. BTZ, but not CFZ, is cytotoxic to microvascular endothelial cells from lung or brain at 100 nM concentration. In human pulmonary microvascular endothelial cell (HPMEC)s, BTZ induces multiple stress pathways: proteotoxic stress, endoplasmic reticulum (ER) stress, and mitochondrial reactive oxygen species (ROS) accumulation. This is associated with activation of apoptotic caspase (CASP)9 (mediator of intrinsic-apoptosis), CASP8 (mediator of extrinsic-apoptosis) and CASP3 (terminal executioner of apoptosis). BTZ-treated HPMECs significantly die and lose vascular barrier function by 24 hours post treatment. These events occur independently of external cues. When we compared 100 nM of BTZ to 250 nM of CFZ (1:2.5 being the ratio of concentration in patients), both compounds elicit comparable cytotoxicity, but elevated ER stress and apoptotic markers are driven by BTZ. Both compounds kill multiple myeloma cells comparably. PBMCs resist drug-mediated changes. Neither inhibitor elicits inflammatory response from HPMECs, MM1.S cells or PBMCs, demonstrating that proteasomal inhibition-mediated death is not inflammatory. As MM is an inflammatory cancer, the natural microvascular environment of patients contains inflammatory cytokines. To model this, we tested the effect of exogenously added tumor necrosis factor alpha (TNF), a key inflammatory cytokine found in MM patients or supernatants from drug-treated MM1.S or PBMCs on HPMECs. Exogenously added TNF or cell-free supernatant from proteasomal inhibitor-treated immune or multiple myeloma cells combined with TNF, further compromises the cellular integrity of HPMECs when compared to drug alone conditions. Physiological levels of TNF enhance stress response in both BTZ- and CFZ-treated cells. This indicates that although proteasomal inhibition is not inflammatory, TNF present in the microvascular environment synergizes with the drugs to compromise endothelial function. Our observations provide an explanation for how microvascular damage potentially underlies tissue injury driven by Bortezomib or Carfilzomib.

## 1 Introduction

The ubiquitin-proteasome pathway maintains cellular homeostasis by degrading abnormal or excess proteins (1). Inhibition of the proteasome results in accumulation of polyubiquitinated proteins, cellular stress buildup and ultimate death (2). Multiple myeloma (MM) is a hematologic malignancy defined by the proliferation of abnormal plasma cells in the bone marrow and overproduction of monoclonal immunoglobulin (3). To meet the demand of high protein turnover, MM cells display high proteasomal activity. Thus, proteasomal inhibitors (PI)s have emerged as key chemotherapy treatment options for this disease (4). The first-in-class PI, Bortezomib (BTZ), was approved in 2003 for relapsed and refractory MM. This became first-line chemotherapy in 2008 (5). BTZ is also approved for mantle cell lymphoma and shows promise in preclinical studies for solid tumors such as non-small cell lung cancer (NSCLC). The second-generation PI, Carfilzomib (CFZ), is an irreversible inhibitor approved in 2012 for advanced MM monotherapy and in 2015 for combination regimens (6). BTZ is administered as intravenous bolus or subcutaneous injection whereas CFZ is administered as intravenous infusion (7, 8). Both drugs get biodistributed to different tissues via blood vessels and microvessels, where they encounter barrier endothelial cells, white blood cells, red blood cells along with circulating myeloma cells (9, 10). Despite their clinical efficacy, the compounds associate with adverse effects, including peripheral neuropathy, cardiovascular and renal toxicities, as well as pulmonary complications (11–15). Currently, there is no clear resolution of how these compounds interact with tissue niches for on-target and off-target effects underlying these pathologies. MM is characterized by heightened cytokine levels and the elevated inflammatory state associates with poor prognosis (16). To benefit therapy and reduce adverse bystander effects, it is crucial that we understand how drug interactions occur in the inflammatory, multi-cellular context of the tissue. MM cells originate from and reside in the bone marrow (17). Some portion of cells escape the bone marrow and circulate in blood, being referred to as circulating plasma cell (CPC)s (18). MM progression and therapy resistance strongly rely on both the bone-marrow niche and CPCs (19). PIs target cancer cells in both bone-marrow and bloodstream (20). The microvessels contain peripheral blood components (including CPCs, immune cells) and are lined by endothelial cells (21). Here, we model the microvascular environment by studying the effect of CFZ and BTZ on endothelial cells, circulating peripheral blood mononuclear cell (PBMC)s and MM cells (MM1.S). These are some of the primary cells found in the microvascular niche.

BTZ is a reversible inhibitor that binds to the catalytic β5/chymotrypsin site of the 26S proteasome (22). Inhibition occurs within one hour of treatment, and activity typically returns to normal within 72 to 96 hours post-administration (23). CFZ irreversibly binds to the N-terminal threonine-containing active sites of the 20S proteasome (6). Both inhibitors lead to protein accumulation, endoplasmic reticulum (ER) stress, mitochondrial stress, reactive oxygen species (ROS) production, unfolded protein response (UPR), cell cycle arrest, and apoptosis in cancer cells (2, 24). Apoptosis can occur via two primary pathways: intrinsic apoptosis triggered by mitochondrial stress and regulated by BCL2 family proteins (25) or extrinsic apoptosis involving caspase (CASP)8 activation driven by death receptors such as TNF receptor 1(TNFR1) (26). Under homeostatic conditions, intrinsic apoptosis is inhibited by anti-apoptotic proteins BCL2, BCLXL, and MCL1. Inhibition of these proteins promotes activation of BAX and BAK, which oligomerize on the mitochondrial membrane, compromising membrane integrity and causing cytochrome c release. This results in CASP9 activation, which subsequently activates CASP3, leading to cell death. BTZ treatment reduces BCL2 expression, triggering intrinsic apoptosis in MM cells (27, 28). BTZ also activates extrinsic apoptosis and stress-responsive autophagy (29, 30). Crosstalk between intrinsic and extrinsic apoptosis occurs when CASP8 truncates BID to yield tBid, that can disrupt mitochondrial integrity. This augments CASP9-dependent CASP3 activation (31). How all of these pathways interact in presence of proteasomal inhibition is unclear. Interestingly, TNF contributes to the BTZ-dependent peripheral neuropathy (32–35), an observation that suggests potential for drug-cell-inflammation interactome in tissue niches. Like BTZ, CFZ induces intrinsic and extrinsic apoptosis as well as autophagy in cancer cells (36–38). Both drugs also drive mitochondrial ROS production in MM cells (39). ROS can activate JNK and p38 MAPK pathways, further promoting intrinsic apoptosis (40, 41). These observations underscore the importance of ER stress, mitochondrial stress, and proteotoxic stress in sensitizing cancer cells to proteasomal inhibitors. Recent evidence also implicates a CASP-independent, ER-stress-dependent death process (paraptosis) in cells treated with PIs (42, 43). When apoptotic CASPs are inhibited, necroptosis is unleashed as an alternate form of programmed cell death (26). Canonical necroptosis involves the binding of TNF to TNFR1 or engagement of other TNF-family death domain receptors, leading to activation of receptor interacting protein kinase (RIPK)1. Activated RIPK1 recruits RIPK3 to form the necrosome, resulting in RIPK3 phosphorylation, which subsequently phosphorylates MLKL and induces cell death. In some non-myeloma cancer cells, BTZ induces RIPK3 polyubiquitination and necroptosis (44). In human MM cell lines CFZ is shown to block necroptotic signal (45). These contradictory studies highlight cell-type specific differences in response to proteasomal inhibition and limitation of assessing a singular cell type, prompting our investigations involving multiple cell types.

Our study is designed to identify interaction patterns of BTZ and CFZ with cancerous and non-cancerous cells of the bloodstream. We identify that BTZ is specifically cytotoxic to microvascular endothelial cells (tested cells from lung and brain). These findings align with previous studies demonstrating effects of BTZ on endothelium (46–48) proteotoxicity on human neurons (11). In contrast, comparable concentration of CFZ does not sensitize microvascular endothelial cells to death. When the two drugs are compared at 1:2.5 ratio of concentration both BTZ (100 nM) and CFZ (250 nM) elicit comparable cytotoxicity even though BTZ-treated cells continue to display elevated ER stress and apoptotic markers. MM1.S cells are comparably susceptible to death caused by either BTZ or CFZ. PBMCs from two donors show resistance to BTZ or CFZ and maintain their anti-apoptotic, pro-survival status. Thus, we have identified three distinct types of responses in three prominent cell types of microvascular environment. When we mimicked the inflammatory environment of the microvasculature, TNF alone or TNF+supernatants from drug-treated immune cells (MM1.S or PBMC) enhance drug-dependent death of endothelial. These results provide novel insights into how the mode of proteasomal inhibition determines the fate in cancerous and non-cancerous cells of microvasculature.

## 2 Materials and methods

### 2.1 Cell culture & reagents

Human pulmonary microvasculature endothelial cells (HPMECs) (49, 50) and human brain microvasculature endothelial cells (HBMECs) ScienCell (Cat#1000) were grown as described before (50). HPMECs are primary cells, HBMECs are modified with Lenti-SV40-Ta virus immortalization kit (Capital Biosciences) (50). Both these cells were cultured in Endothelial cell medium. (ScienCell, Cat# 1001) containing 5% of fetal bovine serum (FBS, Cat# 0025), 1% of endothelial cell growth supplement (ECGS, Cat# 1052), and 1% of penicillin-streptomycin solution. (P/S, Cat# 0503). Multiple Myeloma Cells (MM.1S) were purchased from ATCC (Cat# CRL-2974) and cultured in RPMI 1640 with 1% L-Glutamine (Corning, Cat# MT10104CV) supplemented with 10% Fetal Bovine serum (Gibco, Cat# A5256801), 1% antibiotic penicillin streptomycin strip (Corning, Cat# 30-002-CI), and 1% L-Glutamine (Gibco, Cat# 25030081). Human donor PBMCs were purchased from ALLCELLS,(Donor 1 female; Donor 2, male) and maintained in RPMI 1640 (Corning, Cat# MT10104CV), supplemented with 10% Fetal Bovine serum (Gibco, Cat# A5256801), 1% antibiotic penicillin streptomycin strip (Corning, Cat# 30-002-CI), 1% L-glutamine (Gibco, Cat# 25030081), HEPES 25mM (Sigma Aldrich Cas 7365-45-9) and β-mercaptoethanol 50μM (Bio rad Cat#1610710). All cells were maintained at 37 °C in a humidified 5% CO2 incubator as per respective vendor guidelines. When HPMEC, HBMEC, and MM1.S reached at a cell density of 70% confluency, all experiments were performed. FDA-approved Chemotherapy drugs - Bortezomib (Cell Signaling Technology Cat# 2204), and Carfilzomib (Cell Signaling Technology Cat# 15022) were used for all the assays. Pan CASP inhibitors used were zVAD (oME)-FMK (Cell Signaling Cat# 60332) and zVAD-FMK (Enzo, Cat# ALX-260-020-M005). TNF (Peprotech, Cat# 300-01B-1MG), Cycloheximide (Cell Signaling Technology, Cat# 2112), Staurosporine (Tocris, Cat# 1285) were used as needed for experiments.

### 2.2 Endothelial permeability assessment

Endothelial barrier integrity was assessed by measuring the trans-endothelial electrical resistance (TEER). HPMECs and HBMECs were seeded separately in trans well cell culture inserts (Corning, Cat# 3470) in a 24-well plate. Cells were allowed to grow to 70% confluency before treatment with the indicated drugs. After treatment, cells were monitored over the course 0, 3, 6, 24 hours. Resistance readings were taken using the EVOM3 TEER meter (World Precision instrument, Model EVOM3) as per the manufacturer’s instructions. TEER was calculated by multiplying the resistance by the surface area of the well and reported as a percent change.

### 2.3 Cell viability assays

**Figure 1:**
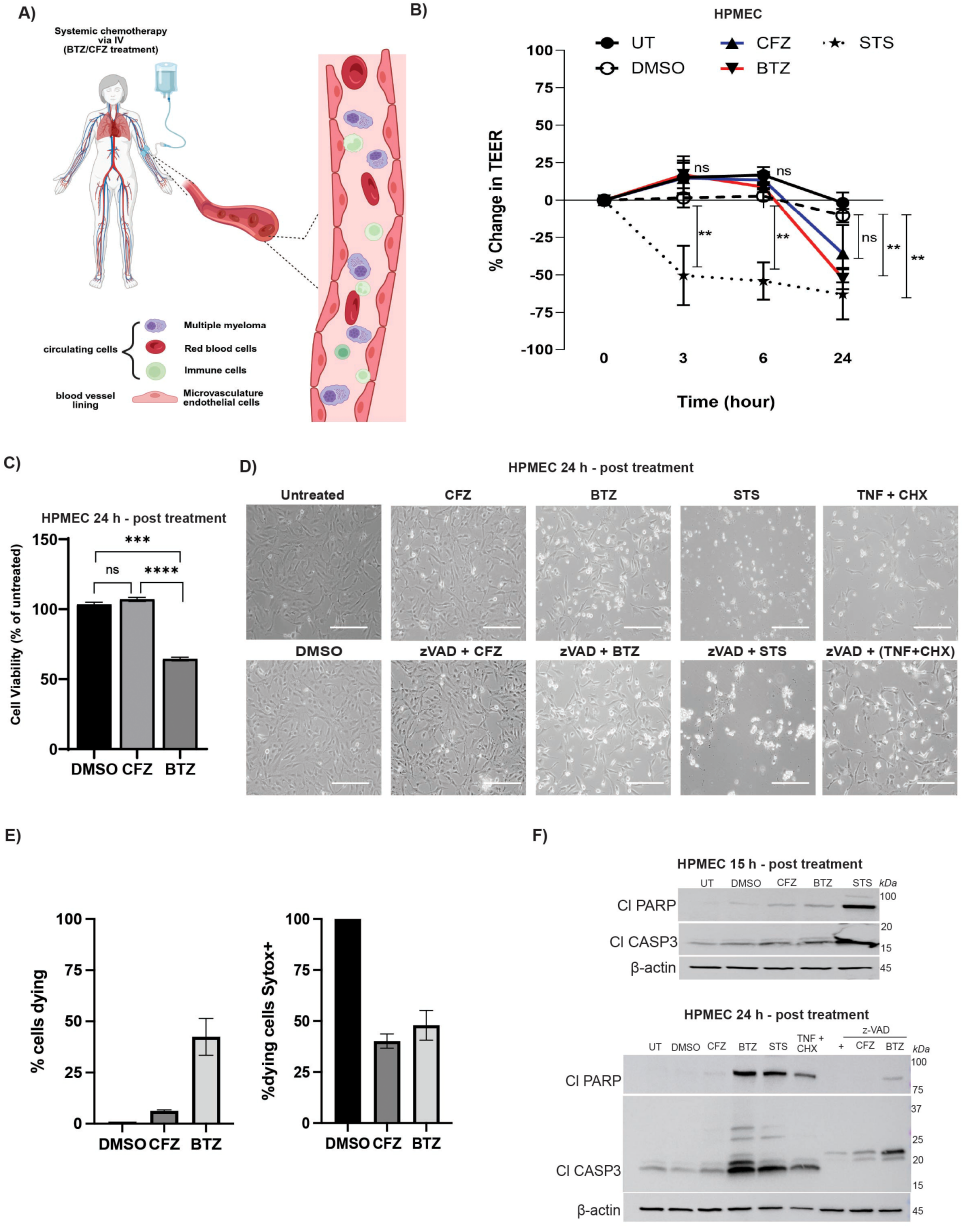
Proteasome inhibitors alter endothelial barrier integrity and induce apoptosis. **(A)** Model figure of multiple myeloma microvascular niche created using BioRender. **(B)** Endothelial barrier integrity was assessed by transendothelial electrical resistance (TEER) in human pulmonary microvascular endothelial cell (HPMEC) monolayers treated with DMSO, Carfilzomib (CFZ, 100 nM), Bortezomib (BTZ, 100 nM) or Staurosporine (STS, 1 μM). Data represent percent change in TEER relative to baseline (error bars represent mean ± SEM, N= 3 independent experiments). Statistical comparison was performed using two-way ANOVA followed by Tukey’s multiple comparisons test. At 24h, DMSO vs. BTZ is **p < 0.01, DMSO vs. STS is **p < 0.01, DMSO vs. CFZ is non-significant (ns); all other time points showed no significant differences for CFZ or BTZ compared to DMSO. DMSO vs STS **p < 0.01 for 3 and 6h. **(C)** Cell viability of HPMEC was assessed based on loss of intracellular ATP after 24h treatment with CFZ (100 nM) or BTZ (100 nM) and DMSO as control. Data represent percentage of viable cells relative to untreated (error bars represent mean ± SEM, N= 6–12 wells from one experiment). Statistical comparison was performed using unpaired, non-parametric Mann-Whitney test. CFZ vs. DMSO is ns; BTZ vs. DMSO is ***p = 0.0001, BTZ vs CFZ is **** p < 0.0001. **(D)** Morphological changes were assessed using brightfield microscopy. Shown is representative (from N= 3 independent experiments) brightfield images of HPMECs treated for 24h with DMSO, CFZ (100 nM), BTZ (100 nM), STS (1 μM), TNF (25 ng/ml) plus CHX (1 μM), or zVAD (20–25 mM) in combination with CFZ, BTZ, STS, or TNF plus CHX. Scale bar=170 μm. **(E)** Cells visible in brightfield images were counted using ImageJ Cell Counter Plugin to detect dying cells (left panel). FITC (SYTOX Green) signal was counted in cells expressing dying morphology (floating, circular morphology, fragmented, blebbed) from microscopy brightfield images merged with FITC (SYTOX Green) fluorescent signal (right panel). Error bars indicate (error bars represent mean ± SEM, from N= 3 images from 3 independent experiments). No significance is indicated as none of the conditions show significance based on unpaired, non-parametric Mann-Whitney test **(F)** Representative (from N= 3 independent experiments) immunoblot analyses shows apoptosis markers in HPMECs treated with DMSO, CFZ (100 nM), BTZ (100 nM), STS (1 μM), TNF (25 ng/ml) plus CHX (1 μM), zVAD, (20–25 μM), or combinations of zVAD with CFZ, BTZ, or STS. Samples collected at 15h (upper panel) or 24h (lower panel) were assessed for Cl-PARP (89 kDa) and Cl-CASP3 (17,19 kDa) with β-actin (45 kDa) as loading control.

Loss of cell viability was measured based on ATP detection using a CellTiter-Glo^®^ 2.0 Cell Viability Assay kit (Promega Corporation, Cat# G9241) and cell integrity loss was measured using SYTOX™ Green Nucleic Acid Stain (Invitrogen™, Thermo Fisher Scientific Cat# S7020). The viability assay based on ATP detection and SYTOX stain was performed as per the manufacturer’s instructions. 10^4^ cells were seeded in each well of tissue culture treated, 96-Well Optical-Bottom Microplate (opaque walls with clear bottom) for fluorescent detection. ATP luminescence and SYTOX fluorescence were measured using Tecan (Model Spark 10M) at 550-570nm and 503/545nm, respectively. SYTOX Green fluorescent signals of the cells were also examined using the FITC channel of Echo Revolve Microscope (Model RVL2-K3). For characterizing alive versus dying cells, we analyzed three independent microscopic images from cells with different treatment conditions (as described in **Figures 1D–E**; **Supplementary Figures 1C, D**; **Figures 5A, B**). Total cells counted (**Supplementary Figure 1D**) include alive and dying cells using brightfield images. Cell counting was performed manually using ImageJ Cell Counter Plugin, Fiji Image J. Alive cells are defined as cells that show distinct periphery, nucleus, cellular organelles in brightfield images (indicated by white arrows in **Supplementary Figure 1D**, image panel). Dying cells are defined as cells that show lack of adherence (floating), rounded morphology, blebbing and fragmented (indicated by yellow arrows in **Supplementary Figure 1D**, image panel). SYTOX + cells were counted from brightfield images merged with fluorescent FITC (SYTOX) signal (indicated by black arrows in **Supplementary Figure 1D**, image panel and fluorescent image in **Supplementary Figure 1C**).

### 2.4 Immunoblot

**Figure 2:**
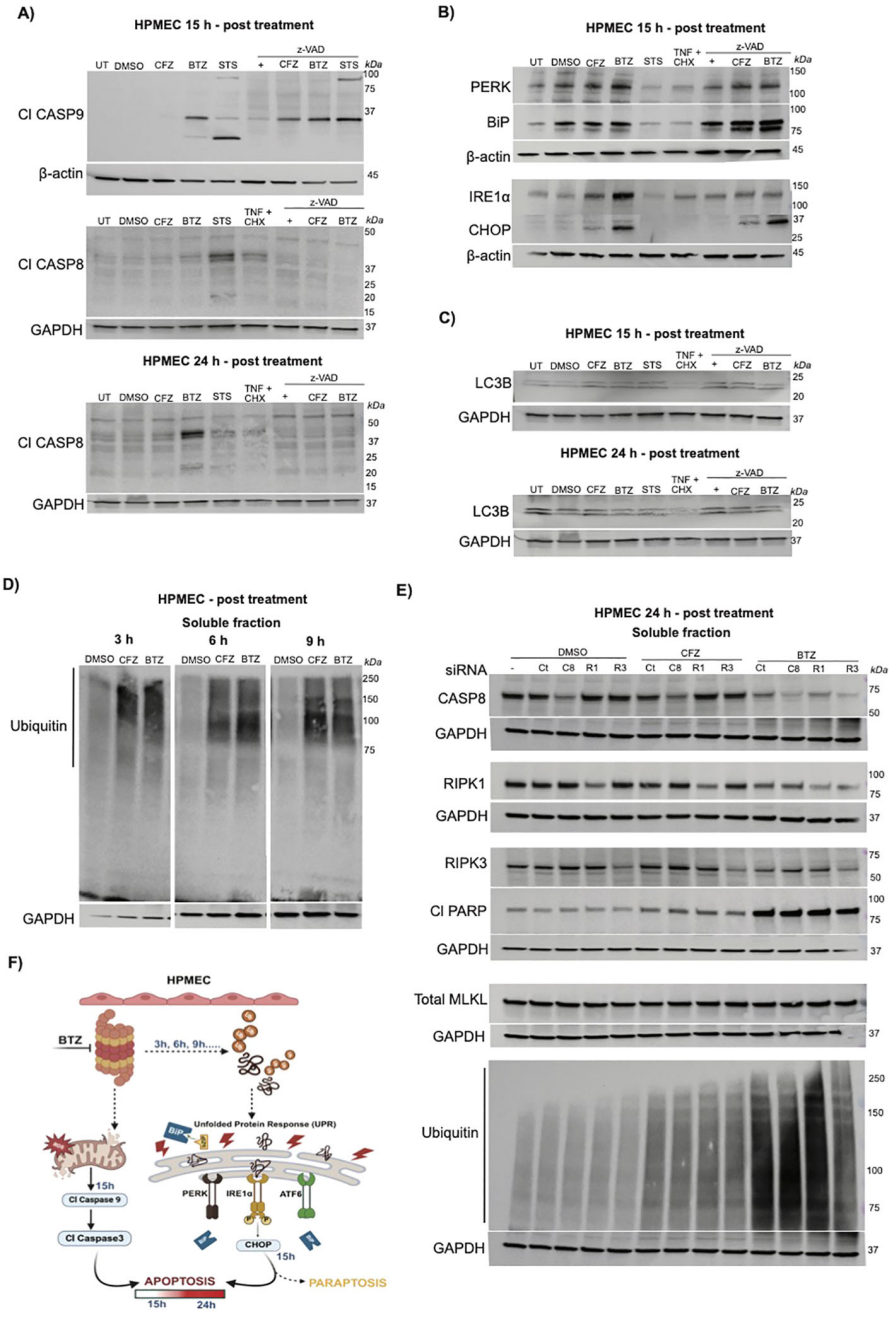
Reversible vs irreversible proteasome inhibitors induce distinct cell-intrinsic mitochondrial, ER, and proteotoxic stress, independent of extrinsic regulators. **(A–D)** Representative (from N= 3 independent experiments) immunoblot analyses show HPMECs treated with DMSO, CFZ (100 nM), BTZ (100 nM), STS (1 μM), TNF (25 ng/ml) plus CHX (1 μM), zVAD (20–25 μM), or combinations of zVAD with CFZ, BTZ, or STS at the indicated time points. **(A)** Immunoblots show intrinsic vs. extrinsic apoptosis markers: Cl-CASP9 (35 kDa) at 15h post treatment (top panel) with β-actin (45 kDa) as the loading control; Cl-CASP8 (43/41/18 kDa) at 15h post treatment (middle panel) and 24h post treatment (bottom panel), with GAPDH (37 kDa) as loading control. **(B)** Immunoblots show ER stress markers, PERK (140 kDa), IRE1α (130 kDa), CHOP (27 kDa), and BiP (78 kDa) in HPMECs at 15h post treatment, with β-actin (45 kDa) as loading control. **(C)** Immunoblots show autophagy marker LC3B-I/II (14/16 kDa) in HPMECs at 15h post treatment (top panel) and 24h post treatment (bottom panel), with GAPDH (37 kDa) a loading control. **(D)** Immunoblots show total ubiquitin accumulation as indication of proteotoxic stress in soluble protein fractions collected at 3, 6, and 9h post treatment. Smears indicate accumulation of ubiquitinated proteins (75kDa to 250kDa), with GAPDH (37 kDa) as loading control. **(E)** Immunoblots detect change in extrinsic regulators (CASP8, RIPK1, RIPK3) in HPMECs transfected with control siRNA (Ct) or targeted siRNA against RIPK1 (R1), CASP8 (C8), or RIPK3 (R3). Cells were treated with siRNA for 48h, followed by 24h treatment with DMSO, CFZ (100 nM), or BTZ (100 nM). Immunoblot shows total CASP8(57 kDa), RIPK1 (76 kDa), RIPK3 (57 kDa), cleaved PARP (Cl-PARP; 89 kDa), total MLKL (54 kDa), and total ubiquitin in soluble fractions with GAPDH (37kDa) as loading control. Images are representative from N= 2 independent experiments for Ct and RIPK1 siRNA with CFZ, and N= 1 with C8 or R3 siRNA knockdown with CFZ or BTZ. **(F)** Schematic representation of cell intrinsic stress (proteotoxic, ER and mitochondrial stress) induced by BTZ in HPMECs created using BioRender.

Immunoblot was done using Triton X-soluble and insoluble extracts from cells; extracts were obtained using two different methods. To obtain cell lysates used in **Figure 2A** (upper panel) and **Supplementary Figure 3B**, after treatment, media was collected from wells into sterile microcentrifuge tubes. This fraction contains the floating dead or dying cells. Tubes were centrifuged at 15000 g for 5 mins at 4 °C to separate media from cells (forming pellet). Cell pellets were kept on ice with 10 µl of complete lysis buffer (Triton X-100 extraction lysis buffer, pH 7.4 [Thermo Scientific Chemicals, Cat# J62289.AK] supplemented with proteases inhibitor cocktail [Roche, Cat# 11836170001] and phosphatase inhibitor cocktail [Roche, Cat# 4906837001] tablets). In parallel, ice-cold complete lysis buffer was added to adherent cell monolayers (held on ice). Cells were then scrapped using a flat-tipped scraper. This lysate was mixed in with the microcentrifuge tube containing cell pellet from detached cells. For all other immunoblots using HPMECs and shown in other figure panels, cells were scraped in treatment media, solution transferred to sterile microcentrifuge tube and centrifuged by quick spin at 15000g for 1–3 mins at 4°C. For suspension cells (MM1.S or PBMC), cells were gently scraped from well and solution transferred to sterile microcentrifuge tube for centrifugation at 15000g for 1–3 mins at 4°C. Cell free supernatant was separated from the cell pellet after centrifugation and complete lysis buffer was added to the pellet. Cell lysates obtained post scraping or centrifugation were shaken for 30 mins at 4°C or maintained on ice for 30 mins followed by re-centrifugation at 15000g for 30 mins at 4°C. This yields the Triton X-soluble cytoplasmic cell lysate solution and Triton X-insoluble pellet fraction containing aggregate proteins as well as membranous components. The soluble lysates were carefully separated from the pellet. Total protein in this soluble cell lysate was quantified using the Bradford’s method (with BSA as a control) according to kit manufacturer’s instructions (Pierce™ BCA Protein Assay Kit, Thermo Fisher Scientific Cat# 23225). For insoluble protein fraction, pellets were sonicated for 10 s at 20 watts in 150 μL of disruption buffer (50 mM Tris pH 6.8, 5% β-mercaptoethanol, 2.75% sucrose and 2% [*w*/*v*] SDS). Soluble lysate samples for immunoblot were prepared using Laemmli SDS sample buffer (Thermo Scientific Chemicals, Cat# J61337.AD). This solution and the Triton-X insoluble fraction solution were boiled at 95°C for 5 minutes immediately prior to loading. Equivalent amounts of protein lysate (30-50 μg) or equivalent volumes of cell lysates (20-30 μl lysed supernatant) were loaded into SDS/PAGE gels (Mini-PROTEAN TGX, 4-20%, Biorad, Cat# 456109) and separated proteins were transferred to PVDF membranes (Bio-Rad, Cat# 1620177). Membranes were then blocked with 5% BSA in TBS-T for 1hr at room temperature and then exposed to primary antibody. Primary antibodies were all dissolved as 1:1000 dilution in blocking buffer. Antibodies used from Cell Signaling technology are: (Cleaved -Cl) Cl-PARP (Cat# Asp214 D64610 5625, RRID: AB_10699459), Cl-CASP3 (Cat# D175 9661, RRID: AB_2341188), Cl-CASP9 (Cat# 20750, RRID: AB_2798848), Cl-CASP8 (Cat# 9496, RRID: AB_561381), LC3B (Cat# 83506, RRID: AB_2800018), PERK, IRE1a, CHOP, BiP (ER Stress Kit Cat# 9956), ICAM1 (Cat# 62133T), RIPK3 (Cat#10188S), CASP8 (Cat# 4790), Ubiquitin (Cat# 20326T), MLKL (Cat# 26539T), BCL2 (Cat#4223T), β-ACTIN (Cat# 4970) and GAPDH (Cat# 8884). ZO1 is from Invitrogen (Cat# 33-9100) and RIPK1 is from BD Biosciences (Cat#610458). Primary antibodies were incubated in at 4 °C, overnight with shaking. After overnight incubation, the membranes were washed three times with 1X TBST. Before expose to secondary antibody. Secondary antibodies used were Anti-Rabbit (Cell Signaling Technology, Cat# 7074) and Anti-Mouse IgG (Cell Signaling Technology Cat# 7076) as 1:2000 dilution. Membranes were incubated with secondary antibody for 1hr at room temperature. Membranes were then washed with 1X TBST three times and developed using ECL western blotting substrate (Biorad, Cat# 1705061) in a KwikQuant Pro Imager (Model D1010) device.

### 2.5 Detection of reactive oxygen species and mitochondria

Cellular ROS was detected using the CellROX™ green Reagent, for oxidative stress detection (Invitrogen™, Thermo Fisher Scientific, Cat# C10444). Briefly, HPMECs were plated on 6 well plates and grown to 80% confluency. Cells were treated for 15–16 hours. After treatment with drugs ROS was measured, 5 μM CellROX green reagent was added to all the wells and incubated for 30 mins at 37 °C followed by 5 mins PBS wash 3X, the cells were then stained with 50 nM MitoTracker™ Dyes for Mitochondria Labeling (Invitrogen™, Thermo Fisher Scientific, Cat# M22425) for 30 mins followed by 5 mins PBS wash 3X, after all the washes cells were fixed with 15 mins in 4% PFA. The fluorescent green and red signals of the cells were examined using Echo Revolve Microscope (Model RVL2-K3).

### 2.6 Cytokine array analysis and ELISA

**Figure 3:**
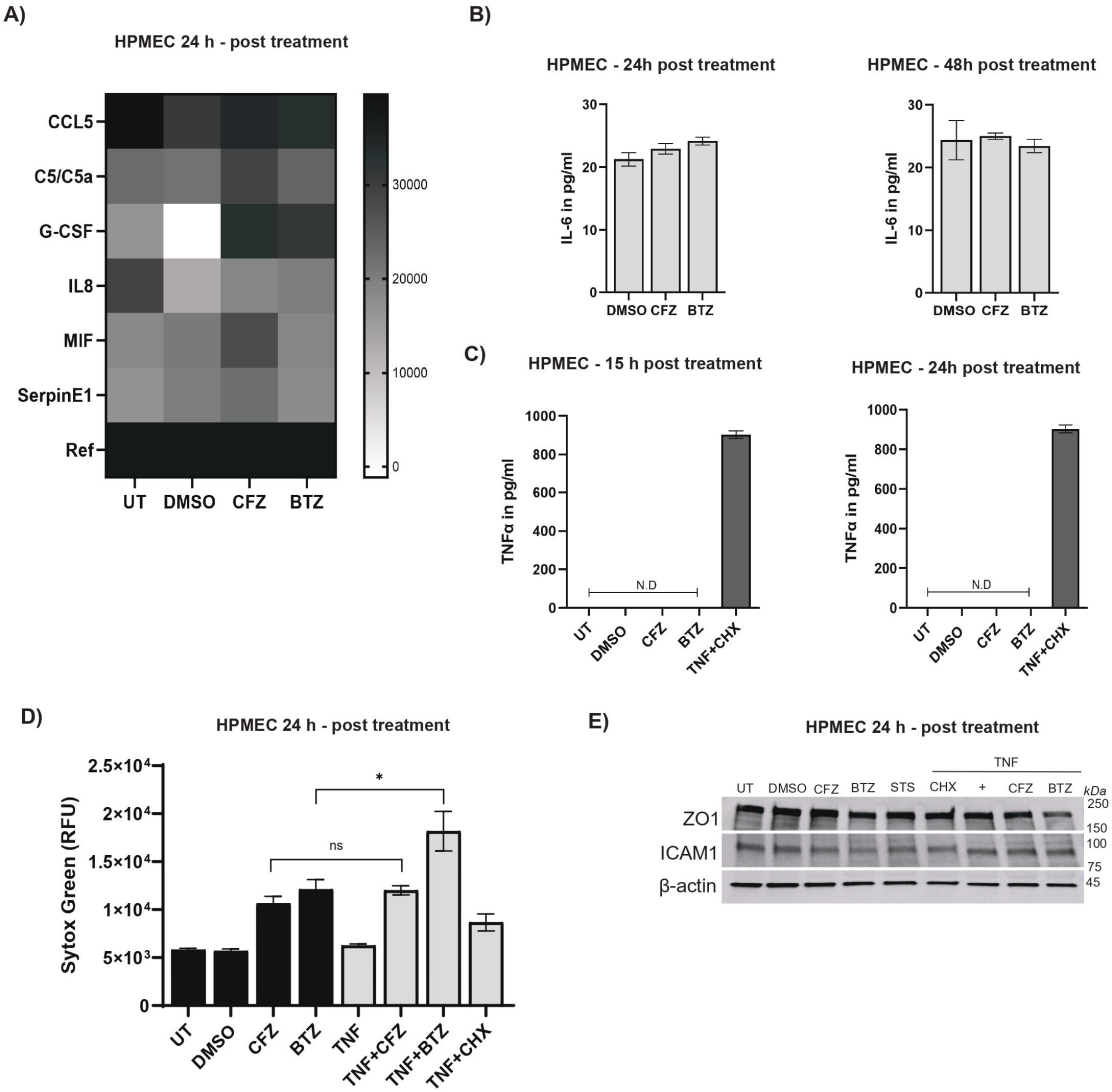
Proteasome inhibitors do not induce inflammation. **(A)** Heatmap representation to show cytokine and chemokine profile in supernatants from untreated (UT), DMSO-, CFZ (100 nM)-, or BTZ (100 nM)-treated HPMECs. Cells were treated for 24h and proteome profile in supernatant was generated using a semi-quantitative dot blot system (Human Proteome Profiler Cytokine Array). For each condition, supernatants were pooled from N= 3 independent experiments. **(B)** IL-6 quantities were measured by ELISA of supernatants from HPMECs treated with DMSO, CFZ (100 nM) or BTZ (100 nM) for 24h or 48h. Statistical comparison was performed using non-parametric Kruskal-Wallis test; no significant differences were observed across DMSO, CFZ, and BTZ at either time point (error bars represent mean ± SEM, N= 3 independent experiments for 24h and N= 2 independent experiments for 48h with three replicates in each experiment). **(C)** TNF-α quantities were measured by ELISA in supernatants from HPMECs left untreated (UT) or treated for 15h or 24h with DMSO, CFZ (100 nM), BTZ (100 nM), or TNF (25 ng/ml) plus CHX (1 μM). TNF was not detected (ND) for any condition except TNF plus CHX. Error bar represent mean ± SEM. All conditions were N= 3 independent experiments. **(D)** Assessment of SYTOX Green uptake in HPMECs left untreated (UT) or treated for 24h with DMSO, CFZ (100 nM), BTZ (100 nM), TNF (25 ng/ml), TNF (25 ng/ml) plus CFZ (100 nM), TNF (25 ng/ml) plus BTZ (100 nM), or TNF (25 ng/ml) plus CHX (1 μM). Statistical comparison performed using the unpaired, non-parametric Mann–Whitney test shows no significant difference for any condition except TNF plus BTZ vs. BTZ (*p < 0.05). TNF plus CFZ vs CFZ is non-significant (ns); error bars represent mean ± SEM, N= 3 independent experiments). **(E)** Representative (from N= 2 independent experiments) immunoblots show appearance of ZO-1 (250 kDa) and ICAM-1 (89,92 kDa) in HPMECs treated for 24h with DMSO, CFZ (100 nM), BTZ (100 nM), STS (1 μM), TNF (25 ng/ml), TNF (25 ng/ml) plus CFZ (100 nM), TNF (25 ng/ml) plus BTZ (100 nM), TNF (25 ng/ml) plus CHX (1 μM) with β-actin (45 kDa) as loading control.

Released cytokine and chemokines were detected by dot blot cytokine array system (Proteome Profiler Human XL cytokine array, R&D systems Cat# ARY005B). To collect supernatants, cell were left untreated or treated as indicated. After treatment, cells were scraped in media and transferred to sterile tubes for centrifugation at 500 g for 5 min at 4 °C. After centrifugation, cell-free supernatants were carefully separated from cell pellet. Supernatants were pooled from three biological replicates for each condition to be included in the cytokine array. Pooled supernatant for each condition was then applied to one membrane and all four membranes (for the four conditions shown) were processed at same time. The experiment was performed according to the manufacturer’s instructions and dot blot membranes were developed using Streptavidin-HRP reagent (provided with kit). Membranes were imaged using (BioRad ChemiDoc Imaging System) as described for immunoblot membranes in section 2.4. Dot blot membrane images were analyzed following methods described before (51). Imaged membranes each had two dots for each protein and six reference dots distributed across the membrane (two dots in each location). This distribution of the reference dots is used to gauge whether the HRP-based development were consistent across the membrane. Integrated density was quantified for each dot detected on each membrane using Adobe Photoshop 2023. For references dots, integrated densities of the six dots (from across the membrane) were averaged. All reference dots within the same membrane displayed comparable densities. Normalization between the groups were done by the following two-step method: Step 1, baseline normalization: The group with the lowest average reference pixel density was used as the baseline for normalization. We designated this as Ref_lowest_ or R_L._ R_L_ was then subtracted from reference spot average density of each membrane. So if membrane 1, 2, 3, had reference spot averages as R_1_, R_2_ and R_3_ respectively, the normalized reference values for the four membranes membrane were (R_1_-R_L_), (R_2_-R_L_) and (R_3_-R_L_) respectively. For membrane giving he value R_L_, this was used as its reference. In **Figure 3A**, these four values are represented in the heatmap as Ref. Step 2, protein signal normalization: For each membrane the integrated density was quantified for two dots of each protein (p) signal visible. This is called Density_protein_ or D_p_. We then normalized the density for each p with the normalized baseline density for that membrane using the following formula. We explain the formula using membrane 1 as an example. For each protein, D_p,normalized_=D_p_- (R_1_-R_L_). D_p,normalized_ for two dots of each protein was then averaged to obtain D_p,final_. D_p,final_ for each protein is plotted in the heatmap shown **Figure 3A**. For quantification of cytokines, sandwich ELISAs were performed using supernatants of untreated or treated (as indicated in Figures) cells as per ELISA manufacturer’s protocols. Kits used were to detect human TNF (R&D systems Cat# DY210) and human IL6 (Cat #DY206). For adherent cells (HPMECs), supernatants were collected as described above in the cytokine array protocol. For suspension cells (MM1.S and PBMCs), wells were gently scraped and cell solutions transferred to sterile tubes to proceed with centrifugation as with HPMECs. 100 µl of each sample was assessed in each well of the ELISA plate. After ELISA, optical density of each well were measured using Chromate plate reader (Model 4300) at 450 nm with reading at 570 nm was subtracted from the 450 nm reading as correction. For calculation, duplicate readings for each standard were averaged to draw the standard curve. Standard curve included the mean absorbance for each standard on the y-axis against the concentration on the x-axis. Concentration of each sample was calculated from the linear-regression equation generated from this standard curve.

### 2.7 siRNA knockdown of CASP8, RIPK1 and RIPK3

Accell SMARTpool siRNAs were used for all knockdown experiments, following the manufacturer’s instructions. Briefly, HPMECs were plated in 6-well plates and incubated overnight at 37 °C with 5% CO_2_. At approximately 40% confluency, cells were treated with 1 µM Accell SMARTpool siRNAs targeting CASP8 (Dharmacon, Cat# E-003466-00-0005), RIPK1 (Dharmacon, Cat# E-004445-00-0005), or RIPK3 (Dharmacon, Cat# E-003534-00-0005). Accell Non-Targeting Control Pool siRNA (Dharmacon, Cat# D-001910-10-20) served as a negative control. Before siRNA treatment, growth media were removed and replaced with Accell delivery media (Cat # B005000-100) containing 1 µM of the respective siRNA. Cells were incubated in delivery media for 48 hours to achieve effective knockdown. After 48 hours, the delivery media were removed and replaced with standard growth media. Cells were then treated with DMSO, CFZ (100 nM), or BTZ (100 nM) for an additional 24 hours. Following treatment, cells were harvested for routine protein extraction and immunoblot analysis.

### 2.8 Conditioned media assay

MM1.S cells were seeded at 0.5 × 10^6^ cells per well in 6-well plates. Human PBMCs were seeded at 1 × 10^6^ cells per well in 6-well plates. Both cell types were treated with DMSO, CFZ (100 nM), BTZ (100 nM), or TNF alone, TNF plus CFZ (100 nM), TNF plus BTZ (100 nM), for 24h. Following treatment, cell-free supernatants were collected and centrifuged at 500 × g for 5min at 4°C. The clarified supernatants were collected and stored at −80°C until use as conditioned media. To assess the effects of MM1.S- or PBMC-derived soluble factors on HPMECs, conditioned media from each treatment group were applied to HPMEC cultures. For the SYTOX Green assay, HPMECs were seeded at 1 × 10^4^ cells per well in 96-well plates. HPMECs were treated with 50 µL media volume containing DMSO, BTZ (100 nM), or CFZ (100 nM) was added to each well, followed by 50 µL conditioned media from MM1.S or PBMC treatment groups, resulting in a final volume of 100 µL while maintaining the final drug concentration of 100 nM. After 24h, cell death was quantified by SYTOX Green fluorescence as described in Section 2.3.

### 2.9 Lactate dehydrogenase assay

To detect lactate dehydrogenase (LDH) released in the supernatant versus associated with cells, 10^4^ cells were seeded in 96 well plate (compatible with the plate reader) in 100 µL of total media volume. The assay was performed according to the manufacturer’s protocol using the CyQUANT LDH Cytotoxicity Assay Kit (Invitrogen™, Thermo Fisher Scientific, Cat# C20300). After treatment with the respective drug groups, 50 µl of conditioned medium from each well of the 96-well plate was transferred to a new clear plate designated as the LDH assay plate. These plates are also compatible with the plate reader. Following manufacturer’s instructions 50 µL of reaction mixture was added to this LDH assay plate containing 50 µL media. The mixture was then incubated for 30 minutes at room temperature. Reaction was stopped by adding 50 µL stop solution before measuring optical density 492 nm with background correction at 630 nm on Chromate plate reader (Model 4300). % cytotoxicity (on y-axis) was calculated based on given manufacturer’s formula: (Experimental LDH Release - Untreated Background)/(Maximum LDH Release Control - Untreated Background) X 100. To quantify LDH associated with cells, we assessed LDH from the treatment plate set aside and containing cells (non-lysed) in remaining 50 µL of media. LDH assay was performed with this plate as described for the supernatant place. Reading from this plate yields the cell-associated LDH activity % max LDH (on y-axis) using the same formula applied for other plate. Negative calculated values were set to zero due to limitations in colorimeter sensitivity and signal detection.

### 2.10 Statistical analysis

**Figure 4:**
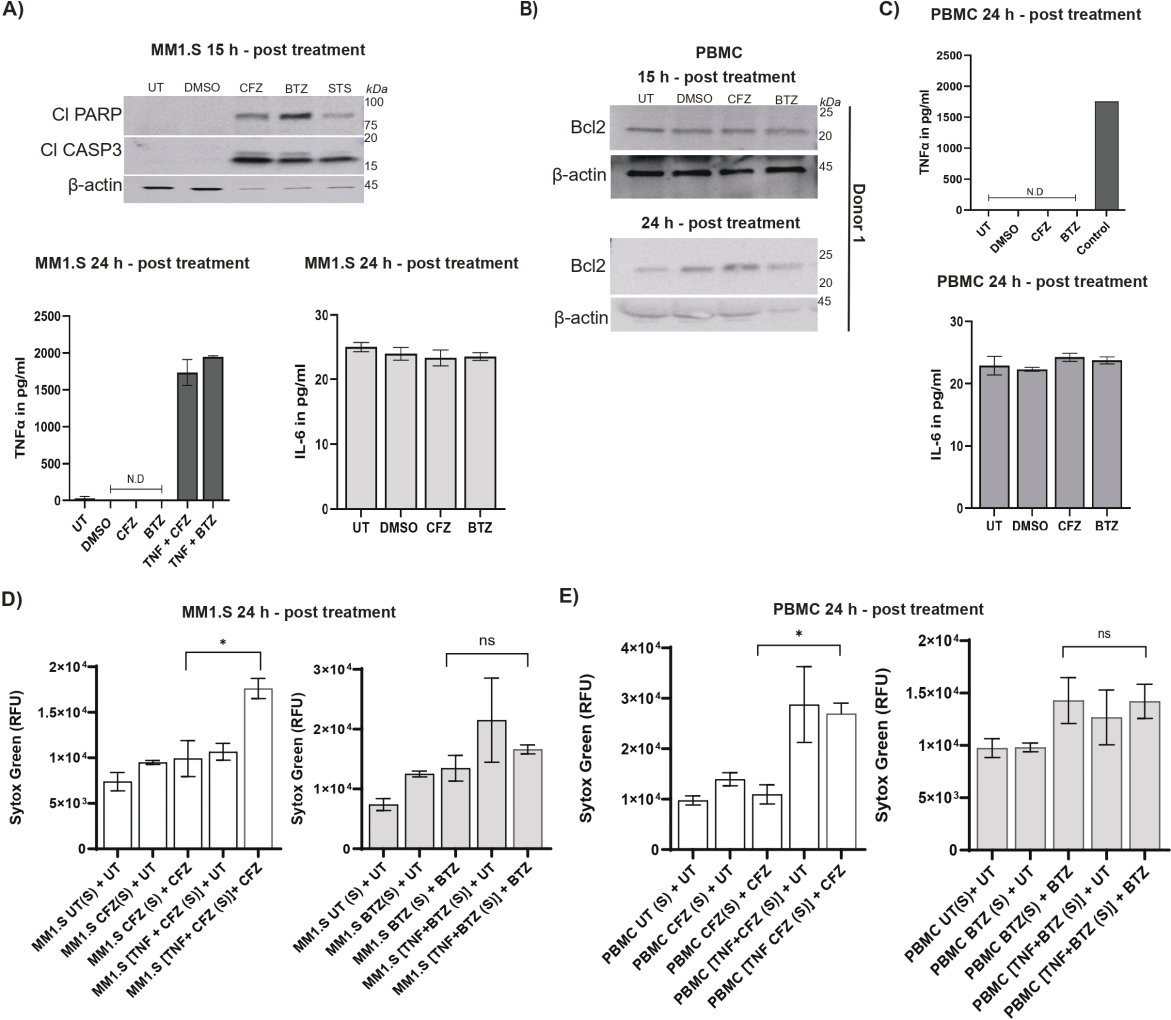
Differential sensitivity of multiple myeloma cells and human PBMCs to proteasome inhibitors and their modulation on endothelial stress. **(A)** Representative (N= 2 independent experiments, upper panel) immunoblots show MM1.s cells left untreated (UT) or treated with DMSO, CFZ (100nM), BTZ (100 nM) and STS (1 μM) for 15h, with Cl-PARP (89 kDa) and Cl-CASP3 (17, 19 kDa), with β-actin (45 kDa) as loading control. Lower panel shows TNF and IL-6 quantities in MM.1S cells left untreated (UT) or treated with DMSO, CFZ (100 nM), or BTZ (100 nM) as measured by ELISA (error bars represent mean ± SEM, N= 3 independent experiments for DMSO, CFZ, BTZ group, N= 1 for TNF plus CFZ or TNF plus BTZ). TNF was not detected (ND) for DMSO, CFZ or BTZ and minimally evident for UT. As expected, conditions with exogenously added TNF (TNF+BTZ or TNF+CFZ) showed TNF quantities; IL-6 quantities are non-significant (ns) in difference between groups when statistics was performed using non-parametric Kruskal-Wallis test. **(B)** Representative (from N= 2 independent experiments) immunoblots show PBMCs (Donor 1) treated with DMSO, CFZ (100nM), or BTZ (100 nM) for the indicated time, with BCL2 (26 kDa) with β-actin (45 kDa) as the loading control. **(C)** TNF and IL-6 quantities are measured by ELISA in supernatant from human PBMCs treated for 24h with DMSO, CFZ (100nM), or BTZ (100 nM) (error bars represent mean ± SEM, N= 3 independent experiments). Statistical comparisons are performed using the non-parametric Kruskal-Wallis test and showed no significant changes in TNF or IL-6 across the groups. Control for TNF plate was supernatants from HPMEC treated with TNF (25 ng/ml) plus CHX (1 µM) for 24h. **(D)** Quantification shows assessment of how soluble factors released from MM1.S cells affect compromise HPMEC integrity. Cells treated for 24h with CFZ (100 nM) or BTZ (100 nM) in the presence (+) of conditioned media/supernatant (S) from MM1.S cells, were assessed for SYTOX Green uptake. On the left, untreated MM1.S supernatant was applied to untreated HPMECs (MM1.S UT (S) + UT), while supernatant from CFZ-treated MM1.S cells applied to untreated HPMECs (MM1.S CFZ (S) + UT) and CFZ-treated HPMECs (MM1.S CFZ (S) + CFZ). TNF + CFZ-treated MM1.S supernatant applied to untreated HPMECs (MM1.S TNF + CFZ (S) + UT) and CFZ-treated HPMECs (MM1.S TNF + CFZ (S) + CFZ). On the right, untreated MM1.S supernatant applied to untreated HPMECs (MM1.S UT (S) + UT), while supernatant from BTZ-treated MM1.S cells applied to untreated HPMECs (MM1.S BTZ (S) + UT) and BTZ-treated HPMECs (MM1.S BTZ (S) + BTZ). TNF + BTZ-treated MM1.S supernatant was applied to untreated HPMECs (MM1.S TNF + BTZ (S) + UT) and BTZ-treated HPMECs (MM1.S TNF + BTZ (S) + BTZ). HPMECs (MM1.S UT (S) + UT) untreated control dataset was used common for both BTZ and CFZ conditions and is displayed in each group (left and right) for comparison. Statistical comparison was performed using unpaired, non-parametric Mann-Whitney test. MM1.S TNF + CFZ (S) + CFZ vs MM1.S CFZ (S) + CFZ is *p<0.05),(MM1.S TNF + BTZ (S) + BTZ vs MM1.S BTZ (S) + BTZ is ns. Error bars represent mean ± SEM, N= 2 independent experiments with three replicates in each except (MM1.S TNF + CFZ (S) + UT), (MM1.S TNF + BTZ (S) + UT), MM1.S TNF + CFZ (S) + CFZ and MM1.S TNF + BTZ (S) + BTZ where error bars represent mean ± SEM from three replicates for each condition in N= 1 experiment. **(E)** Quantification shows assessment of how soluble factors released from PBMCs affect compromise HPMEC integrity. Cells treated for 24h with CFZ (100 nM) or BTZ (100 nM) in the presence (+) of conditioned media/supernatant (S) from MM1.S cells, assessed by SYTOX Green uptake. On the left, untreated PBMC supernatant applied to untreated HPMECs (PBMC UT (S) + UT), while supernatant from CFZ-treated PBMCs was applied to untreated HPMECs (PBMC CFZ (S) + UT) and CFZ-treated HPMECs (PBMC CFZ (S) + CFZ). TNF + CFZ-treated PBMC supernatant was applied to untreated HPMECs (PBMC TNF + CFZ (S) + UT) and CFZ-treated HPMECs (PBMC TNF + CFZ (S) + CFZ). On the right, untreated PBMC supernatant was applied to untreated HPMECs (PBMC UT (S) + UT), while supernatant from BTZ-treated PBMCs was applied to untreated HPMECs (PBMC BTZ (S) + UT) and BTZ-treated HPMECs (PBMC BTZ (S) + BTZ). TNF + BTZ-treated PBMC supernatant was applied to untreated HPMECs (PBMC TNF + BTZ (S) + UT) and BTZ-treated HPMECs (PBMC TNF + BTZ (S) + BTZ). HPMECs (PBMC UT (S) + UT), untreated control dataset was used common for both BTZ and CFZ conditions and is displayed in each group (left and right) for comparison. Statistical comparisons performed using unpaired, non-parametric Mann-Whitney test. PBMC TNF + CFZ (S) + CFZ vs PBMC CFZ (S) + CFZ is *p<0.05; PBMC TNF + BTZ (S) + BTZ vs PBMC BTZ (S) + BTZ is ns. Error bars represent mean ± SEM, N= 2 independent experiments with three replicates in each condition except for PBMC TNF + CFZ (S) + UT, PBMC TNF + BTZ (S) + UT, PBMC TNF + CFZ (S) + CFZ, PBMC TNF + BTZ (S) + BTZ where error bars represent mean ± SEM from three replicates for each condition in N= 1 experiment.

**Figure 5:**
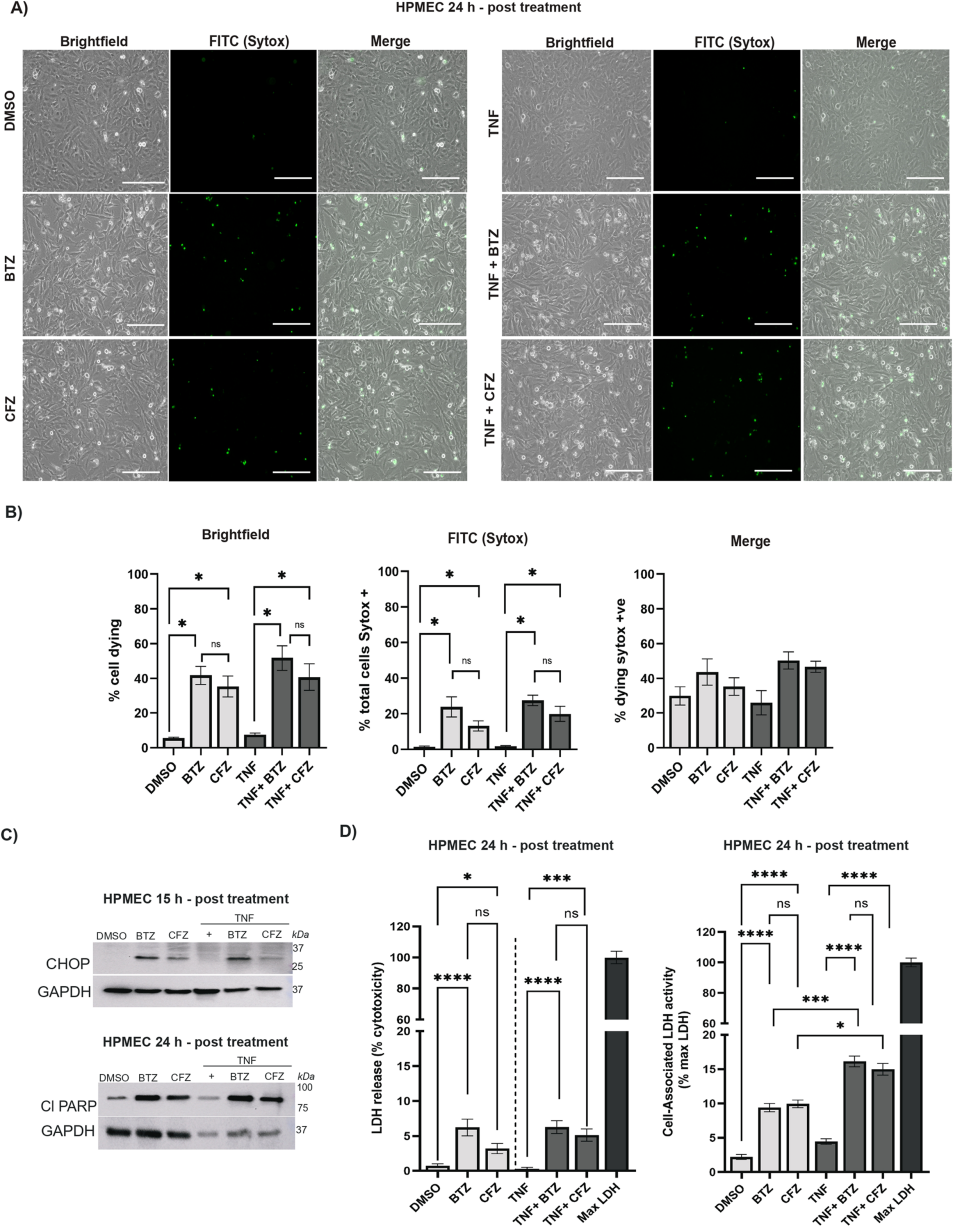
Clinically relevant serum concentrations of proteasome inhibitors and TNF recapitulate TNF-driven modulation of endothelial cell death. **(A)** Morphological changes were assessed using bright-field microscopy. Panel shows representative (from N= 3 independent experiments), brightfield images, FITC (SYTOX Green) signal and merged images (overlay of brightfield and FITC signal) of HPMECs treated for 24h with DMSO (control), BTZ (100 nM), CFZ (250 nM), TNF (1 ng/ml) alone, TNF (1 ng/ml) + BTZ (100 nM), or TNF (1 ng/ml) + CFZ (250 nM). Scale bar=170 μm. **(B)** Cells visible in brightfield images were counted using ImageJ Cell Counter Plugin to detect dying cells (left panel) and FITC punctate signal (middle panel) was counted in cells expressing dying morphology (floating, circular morphology, fragmented, blebbed). Merged images (right panel) brightfield images with FITC signal. Error bars represent mean ± SEM, N= 3 independent images from 3 independent experiments). Statistical comparison performed using unpaired, non-parametric Mann-Whitney test. For the left and middle panels: BTZ vs DMSO is *p < 0.05, CFZ vs DMSO is *p < 0.05, TNF + BTZ vs TNF is *p < 0.05, TNF + CFZ vs TNF is *p < 0.05, BTZ vs CFZ (ns), TNF + BTZ vs TNF + CFZ is ns. No statistically significant difference observed in the right panel. **(C)** Representative (from N= 3 independent experiments) immunoblot analyses show HPMECs treated with DMSO (control), BTZ (100 nM), CFZ (250 nM), TNF (1 ng/ml) alone, TNF (1 ng/ml) + BTZ (100 nM), or TNF (1 ng/ml) + CFZ (250 nM) at the indicated time points. Upper panel: CHOP (27 kDa) at 15h with GAPDH (37 kDa) as loading control; Lower panel: Cl PARP (89 kDa) at 24h with GAPDH (37 kDa) as loading control. **(D)** Cell viability of HPMECs was assessed by LDH assay following 24h treatment with DMSO (control), BTZ (100 nM), CFZ (250 nM), TNF (1 ng/mL) alone, TNF (1 ng/mL) + BTZ (100 nM), or TNF (1 ng/mL) + CFZ (250 nM). Data are presented as percentage cytotoxicity (LDH release; left panel), relative to untreated controls, and percentage of cell-associated LDH activity (% maximum LDH release) (error bars represent mean ± SEM, N= 3 independent experiments). Statistical comparison was performed using non-parametric Kruskal-Wallis followed with Dunn’s multiple comparisons test. For % cytotoxicity (left panel): BTZ vs DMSO is ****p< 0.0001, CFZ vs DMSO is *p= 0.01, BTZ vs CFZ is non-significant (ns); (right panel) TNF vs TNF + BTZ is ****p< 0.0001, TNF vs TNF + CFZ is ***p= 0.0006, TNF + BTZ vs TNF + CFZ is ns, BTZ vs TNF + BTZ is ns, CFZ vs TNF + CFZ is ns. For cell-associated LDH activity (% maximum LDH, right panel): BTZ vs DMSO is ****p < 0.0001, CFZ vs DMSO is ****p< 0.0001, BTZ vs CFZ non-significant is ns; (right panel) TNF vs TNF + BTZ is ****p< 0.0001, TNF vs TNF + CFZ is ****p< 0.0001, TNF + BTZ vs TNF + CFZ is ns, BTZ vs TNF + BTZ is ***p=0.0005, CFZ vs TNF + CFZ is *p= 0.01.

Statistical analyses were performed using GraphPad Prism (Prism version 10). Data are presented as mean ± SEM from at least three independent experiments unless otherwise indicated. Two-independent group comparisons were analyzed using an unpaired, non-parametric, Mann-Whitney test (**Figures 1C, E**, **3D**, **4D, E**, **5B**; **Supplementary Figures 1B, D, 3A**) Multiple-group comparisons were analyzed using the non-parametric Kruskal-Wallis test with appropriate multiple comparisons testing, as indicated in the corresponding figure legends (**Figures 3B, C**, **4B, C**, **5D**) Experiments involving two independent variables were analyzed by two-way ANOVA followed by multiple comparisons testing (**Figure 1B**, **Supplementary Figure 1A**) Statistical details for each experiment are provided in the corresponding figure legends.

### 2.11 Software

Brightfield microscope and fluorescence images were taken using ECHO software, iPadOS 18.6.2. Spark control V1.1SP1 for Tecan readings taken for ATP assay and SYTOX Green assay. Chemiluminescent images of immunoblots were captured using the Kwik Quant Proanalyzer 1.47 and Image lab biorad software. ROS microscope images were enhanced using Adobe Photoshop 2023. ELISA’s raw count was taken using Chromate Manager 6.3. ImageJ (Fiji 64 bit) was used to quantify live, dead, and SYTOX-positive cells from morphological images. All data were assembled using Microsoft Excel and graphed using GraphPad Prism 10. For the cytokine array, pixel intensities were determined using Adobe Photoshop 2023. All figures were assembled using Adobe Illustrator 8. Model cartoons were constructed using BioRender software.

## 3 Results

### 3.1 Proteasomal inhibition compromises microvascular endothelial integrity

BTZ and CFZ both will encounter barrier endothelial cells, immune cells and myeloma cells in the blood vessel or microvessel (9, 10) (**Figure 1A**). To investigate how proteasomal inhibition dictates microvascular function, we treated human pulmonary microvascular endothelial cell (HPMEC)s and human brain microvascular endothelial cells (HBMEC)s with 100 nM of CFZ or BTZ (**Figures 1B–F**; **Supplementary Figures 1A–D**). Pharmacokinetically reported blood concentration for BTZ is 43.7(+/-78.6) ng/ml on low end (52, 53) and for CFZ is 90.2 (+/-84.9) on low end (54). 100 nM of BTZ or CFZ equals to about 38.4 ng/ml or 71.99 ng/ml respectively. We chose 100 nM to assess fundamental differences between the two drugs as this dose is sufficient to completely inhibit the key β5 subunit of the proteasome in MM cells (8, 55, 56). This dose enables us to compare our results to prior preclinical studies (6, 48, 57). To evaluate changes in permeability, we measured transendothelial electrical resistance (TEER) in drug-treated HPMEC or HBMEC monolayers modelling endothelial barrier (**Figure 1B**; **Supplementary Figure 1A**). In this assay, decreased resistance (in % change in TEER) demonstrates increased permeability and loss of barrier function of the monolayer (50). Barrier function is a central biological function of vascular or microvascular endothelium. This regulates passage of cells or other molecules from tissue to blood and vice versa (58, 59). HPMECs treated with vehicle control DMSO, BTZ, CFZ, Staurosporine (STS) all showed decrease in resistance by 24 hours (h) (**Figure 1B**). STS is an inducer of mitochondria-mediated cell death, including intrinsic apoptosis (60) and is used as a positive control for cell death induction. STS-treated cells showed significant permeability within 3h that continued till 24h. This is consistent with visible loss of cells with this treatment at 24h (**Figure 1D**, upper panel). At 24h post treatment, BTZ but not CFZ, significantly reduced resistance compared to DMSO reaching levels similar to STS (**Figure 1B**). These data affirm previous observations demonstrating increased permeability of BTZ-treated HPMECs at doses <100 nM (48). STS treatment exhibited similar pattern of decreased resistance indicating increased permeability in HBMECs (**Supplementary Figure 1A**). At this time, BTZ-treated HPMECs displayed a significant loss of ATP in comparison to DMSO or CFZ (**Figure 1C**). This assay indicated loss of viability of cells. In contrast, CFZ-treated cells did not die significantly when compared to DMSO treatment. HBMECs were significantly more susceptible to both CFZ and BTZ when compared to DMSO. However, BTZ was more effective in killing these cells in direct comparison with CFZ (**Supplementary Figure 1B**). Therefore, independently of the microvascular endothelial cell type tested, BTZ sensitizes cells to death with increased efficiency. Given that HPMECs, display more pronounced differences between BTZ and CFZ (**Figures 1B, C**), we used these cells to further characterize how proteasomal inhibitors dictate cellular signaling.

### 3.2 BTZ triggers apoptotic signal in HPMECs

We treated HPMECs with CFZ or BTZ for microscopic observation. BTZ-treated cells displayed apoptotic morphology including blebbing, appearance of floating circular cellular bodies and loss of adherent cells at 24h post treatment (**Figure 1D** upper panel, **Supplementary Figure 1C** left column). CFZ-treated cells remain attached to the plate surface and were comparable to DMSO-treated or untreated control conditions. To measure loss of cell permeability, we treated cells with cell impermeable dye SYTOX Green (**Figure 1E**; **Supplementary Figure 1C** right column, **Supplementary Figure 1D**). SYTOX incorporates in cells when dying cells lose membrane permeability and binds to nucleic acid. This gives a punctate staining pattern. This is an established indicator of cell death (61, 62). We counted viable cells (adherent, morphologically intact, indicated by white arrows in **Supplementary Figure 1D**) versus dying cells (partially adherent or non-adherent floating, circular morphology, shiny interior without defined organelles, blebbed appearance, indicated by yellow arrows in **Supplementary Figure 1D**). Consistent with the ATP loss assay (**Figure 1C**), ~50% of cells appeared dying in BTZ-treated wells (**Figure 1E**; **Supplementary Figure 1D**). This corresponded with increased SYTOX dye inclusion (marked by punctate staining pattern) in BTZ-treated cells (**Supplementary Figure 1C** right column). Merged image of FITC-SYTOX signal with brightfield images demonstrated that ~30-40% of dying cells included SYTOX signal for both CFZ or BTZ treatment conditions (**Figure 1E**; **Supplementary Figure 1D**). ~20-25% of all cells were SYTOX+ with BTZ treatment. STS was used as control for mitochondrial apoptosis; cytokine TNF plus cycloheximide (CHX) was used as control to activate extrinsic apoptosis, a CASP8-dependent process (61, 63). STS-treated cells exhibited highest loss of adherent cells (**Figure 1D**) which explains loss of fluorescent SYTOX signal (**Supplementary Figure 1C**). TNF+CHX-treated cells displayed loss of adherent cells and SYTOX signal (**Figure 1D**; **Supplementary Figures 1D**). zVAD partially rescued BTZ-, STS- or TNF+CHX-mediated death (**Figure 1D**). We next treated HPMECs with BTZ or CFZ and analyzed cell lysates for appearance of cell death markers (**Figure 1F**). Apoptotic markers tested were cleaved (Cl) PARP and Cl-CASP3 at 15 or 24 h post treatment (**Figure 1F**). As expected, STS induced both Cl-PARP and Cl-CASP3 by 15h. Both CFZ and BTZ showed Cl-PARP and Cl-CASP3 levels comparable to DMSO control at this time (**Figure 1F**, upper panel). However, by 24 h, lysates from BTZ-treated cells displayed high levels of induced Cl-PARP and Cl-CASP3 (**Figure 1F** lower panel). BTZ and STS treatments caused Cl-CASP3 laddering and accumulation of effector fragments at 19 and 17 kDa. TNF + CHX treatment also enhanced quantities of these bands. Pan CASP inhibitor zVAD (61) altered Cl-CASP3 patterns with either CFZ or BTZ. There was no more visible 17 kDa but appearance of 19–20 kDa fragment remained. Another fragment approximately at 22 kDa appeared as well. zVAD binds to catalytic domain of CASPs and prevents autocleavage (64). This dose of zVAD completely prevents Cl-CASP3 accumulation in several cell types (61–63, 65). However this is not confirmed to occur in all cells. The alteration in Cl-CASP3 banding pattern demonstrates that in HPMECs, zVAD at this concentration, prevents accumulation of the 17 kDa fraction, but does not sufficiently prevent 19–20 kDa product formation. As zVAD triggers necroptosis under some conditions (61, 65), we tested for necroptotic markers. But evidence of necroptosis was not detected under any condition. This demonstrates that BTZ dictates CASP-dependent and -independent cytotoxic signals in HPMECs. Recently, a CASP-independent, endoplasmic reticular stress-driven cell death pathway called paraptosis has been described for Bortezomib treatment (43). To determine the type of cell death in HPMECs, we proceeded to characterize the BTZ-induced death.

### 3.3 BTZ drives caspase activation cascade

To explore signals upstream to CASP3 activation, we assessed cleavage of CASP9 and CASP8. CASP9 is a crucial initiator of mitochondrial (intrinsic) apoptosis and is cleaved to render active forms during cell stress conditions such as proteasomal inhibition (66, 67). As expected, STS induced CASP9 cleavage, producing expected 35 kDa, along with a smaller ~20 kDa and larger ~90 kDa products (**Figure 2A**, top panel) in immunoblot. CFZ didn’t induce CASP9 cleavage (**Figure 2A**, top panel), but BTZ induced appearance of the 35 kDa and ~20 kDa products (**Figure 2A**, top panel). At 15 h, these cells did not exhibit CASP3 cleavage (**Figure 1E** upper panel), suggesting that this reversible inhibitor triggers mitochondrial stress leading to apoptosis several hours later. zVAD increased intensity of the 35 kDa cleaved CASP9 form (**Figure 2A**, top panel) revealing potential new insight into zVAD function on CASP9 in HPMECs. CFZ plus zVAD treated cells displayed accumulated Cl-CASP9 but no cell death. Prior studies show that zVAD can partially prevent CASP9 autocleavage by inducing conformational changes. This prevents formation of mature CASP9 (68, 69). We next evaluated extrinsic apoptotic markers via immunoblot detection of Cl-CASP8 (**Figure 2A** middle and bottom panels). At 15 h, Cl-CASP8 appeared in STS-treated cells only (two expected products ~43 kDa, ~41kDa and ~18 kDa) (**Figure 2A** middle panel). By 24 hours, STS-induced CASP8 cleavage was no longer evident (**Figure 2A** bottom panel), likely due to cell loss (**Figure 1F**). TNF + CHX treatment exhibited accumulation of 43 and 41 kDa product at 15 h, but no cleaved forms of CASP8 were detected at 24 h. BTZ treatment lysates exhibited Cl-CASP8 bands only at 24 h but not at 15 h (**Figure 2A** middle and bottom panels). This demonstrates that BTZ triggers a CASP9→CASP8→CASP3 cascade in HPMECs. Therefore, in this setting mitochondrial (intrinsic) apoptotic signal dictates CASP8-dependent (extrinsic) apoptosis.

### 3.4 BTZ dictates ER stress

In cancer cells, PIs drive parallel mitochondrial and ER stress pathways, leading to intrinsic apoptosis as well as paraptosis (42, 43, 70). In human umbilical vein endothelial cells, 750 nM of CFZ fails to elicit ER stress (71). To test how CFZ compares to BTZ in HPMECs, we analyzed ER stress markers via immunoblotting at 15 h post treatment (**Figure 2B**). CFZ induced appearance of CHOP over DMSO treatment (**Figure 2B**). BTZ induced a further elevation in levels of CHOP over CFZ. CHOP is a key ER stress marker induced via PERK-, IRE1-, or ATF6-mediated pathways (72). PERK activated by unfolded proteins, phosphorylates eIF2α, leading to ATF4-driven *Chop* expression (72). In the IRE1 pathway, unfolded proteins trigger IRE1α autophosphorylation, activating XBP-1, which upregulates apoptotic genes, including *Chop* (73). PERK was detected in unmanipulated and DMSO-treated control cells, with no significant elevation under CFZ or BTZ conditions (**Figure 2B**). IRE1α was detected in unmanipulated or DMSO-treated cells with a gradation effect of CFZ<BTZ (**Figure 2B**). This shows that in HPMECs, reversible proteasomal inhibition by BTZ induces increased IRE1α➔CHOP➔ER stress pathway when compared to CFZ at same concentration. This parallels CASP9 and CASP8 activation as well as cell death (**Figures 1F**, **2A**). zVAD did not eliminate increased CHOP levels in BTZ-treated cells (**Figure 2B**) but zVAD reduced IRE1α levels in all conditions (**Figure 2B**). Therefore, the observed ER stress proceeds in CASP-independent and CASP-dependent processes. To determine whether CFZ and BTZ differ further upstream in the ER stress pathway, we analyzed BiP (**Figure 2B**), a chaperone protein that acts as a primary ER stress sensor by interacting with the luminal domains of UPR proteins IRE1α or PERK (74). DMSO, CFZ and BTZ both induced comparable BiP expressions (appearing as a doublet band) over unmanipulated cells (**Figure 2B**). zVAD, with or without drug treatment, further enhanced the doublet. This highlights a fundamental role of CASPs in maintaining physiologic levels of ER stress proteins in these cells microvascular cells. Prior studies also implicate CASPs upstream to ER stress (75). CASP inhibition, even in absence of a drug, dysregulates that physiologic balance. In conclusion, we show that BTZ but not CFZ, specifically induces the IRE1α-CHOP axis, rather than a generalized UPR activation without altering protein chaperoning inside the ER. ER stress in BTZ-treated cells shows contributions from CASP-dependent and -independent signals.

### 3.5 BTZ drives mitochondrial stress in HPMECs

We next examined mitochondrial stress (76) (**Supplementary Figure 2A**) by assessing accumulation of reactive oxygen species (ROS) in the organelle. For this assay we used fluorescent dyes mitotracker (red) and anti-ROS dye (green) (**Supplementary Figure 2A**). Untreated cells showed diffused red staining indicative of active mitochondria (**Supplementary Figure 2A** first row). DMSO-treated cells showed a similar pattern (**Supplementary Figure 2A** second row). We used hydrogen peroxide as a positive control for ROS production (**Supplementary Figure 2A** fifth row) and detected an elevated green (ROS) signal along with reduced spread of Mitotracker (red). This indicates mitochondrial shutdown. When we compared BTZ to CFZ, we found that BTZ drives visibly more ROS green signal and punctate Mitotracker red signal (**Supplementary Figure 2A** third and fourth rows). Punctate Mitotracker signal is indicative of mitochondrial stress and loss of membrane potential, disrupting the even spread of Mitotracker. Notably, ROS accumulation localized in regions where the mitochondrial membrane potential and structural integrity were lost (**Supplementary Figure 2A**, fourth row merged image). To determine contribution from autophagy, we assessed LC3B levels at 15 h (**Figure 2C** top panel) or 24 h (bottom panel) post treatment. LC3B was induced with CFZ, BTZ or STS by 15 h but all treatment conditions had comparable levels. This rules out autophagy involvement in this BTZ versus CFZ cytotoxicity. Our findings confirm that BTZ induces mitochondrial stress by increasing ROS production and disrupting mitochondrial membrane integrity. Thus, 100 nM of BTZ induces cell intrinsic stress via the ER and mitochondria in microvascular endothelial cells.

### 3.6 BTZ and CFZ differentially induce proteotoxic stress

When proteasomal function is inhibited, cells display proteotoxic stress, indicated by accumulation of ubiquitinylated proteins (77–79). We evaluated the accumulation of total ubiquitin levels in the cytosolic protein (Triton-X-soluble) and aggregate protein (Triton-X-insoluble) fractions of the cells (**Figure 2D**, **Supplementary Figure 2B**) (61, 80) at 3, 6 and 9h post treatment. These time points are chosen as accumulation of ubiquitinylated proteins is an early event following proteasomal inhibition. Ubiquitination is a primary signal for protein degradation; but in presence of PIs ubiquitinylated proteins do not degrade and accumulate often forming detergent-insoluble aggregates (81–83). In the cytosolic fraction, both drugs displayed increased accumulation of total ubiquitin over DMSO treatment (**Figure 2D**), even though the patterns of accumulation were distinct. In the triton-X insoluble fraction, BTZ-treated conditions exhibit enhanced total ubiquitin signal (**Supplementary Figure 2B**) compared to CFZ at all time points. Therefore, BTZ induces higher proteotoxic stress compared to CFZ from few hours after treatment. This enhanced accumulation of unfolded, ubiquitinylated proteins likely drives increased IRE-1α-CHOP pathway in BTZ-treated cells (**Figure 2B**). Cellular IRE-1α level is known to increase with increased accumulation of unfolded or misfolded proteins (84, 85). IRE-1α further activates signaling that induce expression of CHOP (72). ER stress overall also drives mitochondria-dependent CASP activation (76). Therefore, we conclude that sequential proteotoxic stress➔ER and mitochondrial stress➔apoptosis drives BTZ-mediated cytotoxicity in HPMECs.

### 3.7 Extrinsic cell death processes are dispensable for BTZ-induced death signal

Activated CASP8 is the mediator for extrinsic apoptosis (86) and RIPK3 drives necroptosis (87). RIPK1 associates with both CASP8 and RIPK3 to aid in extrinsic apoptosis or necroptosis (61, 65, 80). A previous study identified that at 500 nM concentration, in Jurkat or H9 cancer cells, BTZ drives necroptosis (44). To evaluate how extrinsic cell death pathways contribute to BTZ-mediated cytotoxicity in HPMECs, we knocked down CASP8, RIPK1 or RIPK3 via siRNA (**Figure 2E**) and tested for response to 100 nM CFZ or BTZ. Immunoblot analysis of DMSO-treated lanes for control, CASP8, RIPK1 and RIPK3 revealed the efficiency of knockdown for each protein (**Figure 2E**). As expected (**Figure 2A**), 24 h of BTZ treatment exhibited enhancement in accumulation of apoptotic marker Cl-PARP when compared to CFZ treatment. Knockdown of any gene did not alter this level, indicating that CASP8, RIPK1 and RIPK3 are dispensable for BTZ-induced apoptotic signal. Phosphorylated MLKL (marker of necroptotic pathway) was not detected and total MLKL levels remained unperturbed through all conditions. Thus, necroptosis also does not contribute in our reported BTZ effects on these cells. Interestingly, by this time point, BTZ treatment starkly enhance accumulation of total ubiquitin demonstrating an accumulation of cytosolic ubiquitinylated proteins. In cancer cells, 500 nM of BTZ induces polyubiquitination of RIPK3 (44). Consistent with those prior observations, we noticed a reduction in total ubiquitin signal in RIPK3 siRNA knockdown conditions treated with BTZ. In combination, we show that HPMECs respond to BTZ by triggering cell intrinsic stress and apoptotic signaling without primary roles of CASP8, RIPK1 or RIPK3. Even though necroptosis is not associated, RIPK3 contributes to the BTZ-driven proteotoxic load.

### 3.8 BTZ treatment of HPMECs is not inflammatory

Some studies suggest that BTZ induces immunogenic cell death (28), and drives alternations in inflammatory response (88). Importantly, MM is an inflammatory cancer and elevated levels of inflammatory cytokines (including TNF) are hallmarks of disease (16, 89–91). Elevated TNF is detected in the bone marrow microenvironment (89, 92) as well as sera of patients (89, 93). TNF associates with poor prognosis (89, 93) and synergizes with BTZ to dictate tissue injury (33–35, 94, 95). In the tumor microenvironment, TNF is thought to increase permeability of bone marrow endothelial cells allowing increased migration of the cancer cells (90). But, there is much gap in knowledge in how this cytokine or other inflammatory factors impact microvascular cells. To address this, we first characterized microvascular inflammation via semi-quantitative cytokine array and quantitative ELISA. We assessed cytokine and chemokine release from DMSO, CFZ or BTZ treated cells at 15, 24, or 48 h post treatment (**Figures 3A–C**). For the cytokine array we pooled cell-free supernatants (media) from three biological replicates for each of the following conditions: unmanipulated or untreated (UT), DMSO-, CFZ-, and BTZ-treated cells (**Figure 3A**). The heatmap revealed that in each condition cells had a distinct inflammatory profile. Unmanipulated cells showed detectable levels of chemokines CCL-5, C5a, cytokines IL-8, G-CSF, chemokine-like cytokine MIF, and serine protease inhibitor Serpin E1. DMSO-treated cells showed a reduction in CCL-5, C5/C5a, G-CSF, IL-8 and an increase in MIF as well as SerpinE1 indicating that DMSO alters the threshold inflammatory signaling of these cells. Media from CFZ-treated cells displayed an increase in CCL5, C5/C5a, GM-CSF, IL-8, MIF, and Serpin E1 when compared to DMSO. Media from BTZ-treated cells displayed an increase in CCL5, GM-CSF, IL-8 and decrease in MIF, Serpin EI with no visible difference in C5/C5a when compared to DMSO. Media from BTZ-treated cells displayed an overall decrease in CCL5, C5/C5a, GM-CSF, MIF, Serpin EI when compared to CFZ. Only IL-8 appeared elevated in BTZ condition over CFZ. These findings showing are not surprising, as BTZ is known modulate the inflammatory response by downregulation of NFκB transcriptional activity (96). We next quantified production of cytokines TNF and interleukin (IL)-6. Both TNF and IL-6 are pro-myeloma cytokines, detected at high levels in patients and contribute to angiogenesis (97, 98). Neither CFZ, nor BTZ elicited production of IL-6 (measured at 24 or 48h post treatment, **Figure 3B**) or TNF (measured at 15 or 24h post treatment, **Figure 3C**) above DMSO control. No detectable levels of TNF were observed in any treatment group at the indicated time points (**Figure 3C**). TNF + CHX control showed detectable TNF as expected. Cell-extrinsic TNF engages with TNFR1 as a primary signaling mechanism for CASP8 cleavage (61, 62). The absence of TNF in supernatant demonstrates that in HPMECs, BTZ-driven cell intrinsic stress mediates the CASP8 cleavage independently of external cue.

### 3.9 Exogenous TNF with BTZ increases loss of HPMEC integrity

We next assessed the response of CFZ- or BTZ-treated HPMECs to TNF, that is expected to exist in the bloodstream of patients (89, 93). We added exogenous TNF to drug treatment and quantified SYTOX Green signals (**Figure 3D**). As demonstrated in **Figure 3**; **Supplementary Figure 1**, SYTOX signal is an indicative marker of loss of cellular integrity. Exogenous TNF significantly enhanced this signal in BTZ-treated cells over BTZ only condition (**Figure 3D**). This indicates that TNF-TNFR1-driven signal will synergize with BTZ-induced cell stress in the inflammatory environment of the microvasculature. We next investigated expression levels of ZO-1 and ICAM-1. ZO-1 is a tight junction-associated protein and regulates expression of transmembrane junction proteins including VE-Cadherin (99). ZO-1 is a crucial regulator of endothelial permeability. ICAM-1 is a monocyte adhesion molecule on vascular or microvascular endothelial cells induced by inflammatory cytokines (100). There were no significant changes in ZO-1 or ICAM1 in BTZ, CFZ, STS, TNF+CHX, or TNF+CFZ conditions (**Figure 3E**). TNF+BTZ lane displayed a tending reduction in ZO-1 levels. Reduced ZO-1 is a marker of acute endothelial damage (101). Thus 100 nM of BTZ induces non-inflammatory, cell intrinsic stress and loss of cellular function independently of contributions from extrinsic death players (**Figures 1**, **2**); TNF enhances this damage but does not influence BTZ-associated vascular inflammatory marker ICAM-1. In combination these data emphasize that even though BTZ dictates intracellular stress independently of any external cues, in the microvascular environment it will synergize with inflammatory cues to amplify adverse pathology.

### 3.10 PI-treated multiple myeloma cells succumb to non-inflammatory cell death

To find the source of inflammation, we assessed the response of MM cells exposed to CFZ, BTZ, STS and DMSO (**Figure 4A**; **Supplementary Figure 3A**). Both CFZ and BTZ killed human MM (MM1.S) cells compared to vehicle control DMSO within 12 hours of treatment. This was measured by loss of ATP (**Supplementary Figure 3A** left panel). Immunoblot analysis revealed that both BTZ and CFZ trigger PARP and CASP3 cleavage comparably; STS also drove a similar response (**Figure 4A** top panel). zVAD eliminated appearance of both Cl-PARP and Cl-CASP3 demonstrating that in these cancer cells, this death is CASP dependent (**Supplementary Figure 3B**). BTZ alone or TNF+ CFZ/BTZ reduced quantities of levels of antiapoptotic protein BCL-2 (102) (**Supplementary Figure 3C**). MM1.S cells treated for 24h exhibited a different pattern of death. Even though both CFZ and BTZ still induced significant cell death, the overall viability of cells increased (**Supplementary Figure 3A** right panel). This is likely due to doubling of the cancerous cells remaining from 12h and decay of BTZ or CFZ. To address whether MM1.S cells undergo inflammatory changes when exposed to BTZ or CFZ, we quantified TNF and IL-6 production from these cells at 24h post treatment (**Figure 4B** lower panels). IL-6 production was comparable between all conditions; TNF was below level of detection. TNF+BTZ and TNF+CFZ are assay controls used to establish the validity of the assay. These data indicate that even though MM cells are highly susceptible to proteasomal inhibition-induced death, cells do not die in an inflammatory manner. Additionally, they are not likely to be primary producers of inflammatory cytokines when exposed to CFZ or BTZ.

### 3.11 Peripheral blood mononuclear cell are not susceptible to proteasomal inhibitor-mediated cytotoxicity

PBMCs consist of T cells (~70%), B cells (~15%), monocytes (~5%), dendritic cells (~1%), and natural killer (NK) cells (~10%) (103). Leukocyte trafficking from blood to tissues is a primary function of the endothelium and T cell interaction with the endothelium is a primary event in inflammation (104). Thus, we selected this immune cell population to account for any potential T cell interaction with the endothelium. The cells were from two different donors. PBMCs did not exhibit markers of cell death and maintained steady levels of BCL-2 at 15, 24 h and 48 post treatment (**Figure 4B**, **Supplementary Figure 3D**). In contrast to MM1.s or PBMCs, HPMECs did not display detectable levels of BCL-2 (**Supplementary Figure 3D**). This indicates that proteasome inhibitors do not activate HPMECs as low BCL-2 is a characteristic feature of resting endothelium in the tissue (105–107). PBMCs also did not produce inflammatory cytokines TNF or IL-6 (**Figure 4C**).

### 3.12 Crosstalk of MM.1S cells and PBMCs with HPMECs

HPMECs, MM1.S or PBMCs all displayed a common non-inflammatory pattern following proteasomal inhibition even though distinct patterns of cell death occurred in each cell type. Thus, proteasomal inhibition does not cause overall inflammation in the microvascular milieu, but BTZ-treated cells can respond to TNF (**Figure 3D**). To determine whether HPMECs respond to soluble factors released from blood-borne cells along with TNF, we first treated MM1.S cells or PBMCs with CFZ, BTZ, TNF+CFZ or TNF+BTZ. Addition of TNF is to mimic MM tissue environment (93). The cell-free supernatants were collected and then applied on HPMECs (**Figures 4D, E**). HPMECs were left untreated or treated with either drug. Supernatants from MM1.S with or without CFZ treatment did not alter the cellular integrity of untreated or CFZ-treated HPMECs (**Figure 4D** left panel). Supernatant from MM1.S cells treated with TNF+CFZ significantly enhanced cell permeability of HPMECs with CFZ. Supernatants from untreated, BTZ-treated or TNF+BTZ-treated MM1.S cells did not alter the cellular integrity of HPMECs under any condition (**Figure 4D** right panel). PBMCs showed a similar pattern (**Figure 4E**). Supernatant from TNF+CFZ-treated PBMCs significantly enhanced HMEC cell permeability when they were treated with CFZ (**Figure 4E** right panel). Supernatants from untreated, CFZ-treated or TNF+BTZ-treated PBMC did not sensitize HPMECs (**Figure 4E** right panel). In combination, these data show that neither multiple myeloma cells nor PBMCs produce soluble factors (in presence or absence of proteasomal inhibitors) that influences untreated endothelium. TNF present in system directly accentuates BTZ-mediated endothelial effects (**Figure 3D**). Endothelium is also responsive when it is undergoing proteasomal inhibition by CFZ in presence of TNF+other factors produced by the cancerous or non-cancerous immune cells. It is possible that as CFZ-treated HPMECs were not stressed (**Figures 1**, **2**), they had more available cells to display enhanced signs of damage (**Figure 4**) or CFZ specifically enhances production of factors (e.g. extracellular vesicles) by the MM or immune cells that synergize with TNF for HPMEC death. BTZ-treated HPMECs were already stressed (**Figures 1**, **2**) and prone to synergy with TNF (**Figure 3**). The remaining alive cells likely undergo death following TNF+supernatant treatment, before SYTOX incorporation could be measured. In conclusion, these data clearly show external TNF (a hallmark cytokine present in multiple myeloma patients) enhances cytotoxicity of PI (independently of mode of action) on HPMECs. These observations provide a fundamental clarification of why PIs combined with anti-inflammatory steroids show an efficient outcome in patients compared to drug alone (108, 109).

### 3.13 Comparative endothelial cytotoxicity of bortezomib and carfilzomib in relation to clinical pharmacokinetics

According to FDA labeling, intravenous BTZ dose of 1.3 mg/m² in cycle 1 of treatment results in mean maximum plasma concentrations (Cmax) of 112 ng/ml(291 nM, SD 122) (110) and https://www.accessdata.fda.gov/drugsatfda_docs/label/2017/205004s000lbl.pdf. In contrast, CFZ administered at 20 mg/m² in Cycle 1 achieved a Cmax of 528 ng/mL (734 nM, SD 406) and https://www.accessdata.fda.gov/drugsatfda_docs/label/2012/202714lbl.pdf. Pharmacokinetically, reported low-end plasma concentrations were 43.7 ng/mL (± 78.6) for BTZ and 90.2 ng/mL for CFZ (54, 110). Thus, BTZ effective concentration is about 2.5-fold lower than CFZ. To contextualize BTZ versus CFZ with clinically relevant pharmacokinetics, we compared 100 nM of BTZ to 250 nM of CFZ (**Figures 5A–D**). To make these studies physiologically relevant, we also added 1 ng/ml TNF in these settings as in patients, highest record TNF levels are around 1-1.5 ng/ml (89, 93, 111). Previously, we used 25 ng/ml of TNF (**Figures 3**, **4**). Brightfield morphology and punctate SYTOX signal indicating compromised membrane integrity (**Figure 5A**) showed comparable changes between BTZ (100 nM) and CFZ (250 nM). Quantification of cell death based on morphology and SYTOX positivity (explained in **Figure 1**; **Supplementary Figure 1**) confirmed comparable cytotoxicity between BTZ and CFZ in presence or absence of TNF (**Figure 5B**). We next tested how apoptotic and ER stress pathways are induced in this set. At 15 h post-treatment, 100 nM BTZ continued to induce CHOP levels higher than 250 nM CFZ (**Figure 5C**, upper panel). When we assessed for induction of apoptotic signal, Cl-PARP (**Figure 5C**, lower panel) signal appeared higher in BTZ when compared to CFZ condition. TNF (1 ng/ml) did not have any effect on CHOP. When we compared loading control GAPDH to samples with or without TNF, the latter group displayed much reduced GAPDH levels. However, the cleaved PARP levels were comparable in TNF+BTZ or TNF+CFZ to BTZ or CFZ only. This indicates that physiologic levels of TNF enhance apoptotic signal in CFZ- or BTZ-treated HPMECs. Lactate dehydrogenase (LDH) is a cytoplasmic enzyme that is released from dying cells. LDH release is a marker of cytotoxicity and cell death(112). We quantified released LDH and cell-associated LDH activity to test how the drugs individually or in presence of TNF induce cytotoxicity at the different doses (**Figure 5D**). We detected comparable LDH release between BTZ and CFZ in presence or absence of TNF (**Figure 5D**, left panel). When we measured cell associated LDH, this pattern continued between BTZ and CFZ groups (**Figure 5D**, right panel). However, TNF+BTZ or TNF+CFZ displayed significantly elevated cell-associated LDH when compared to BTZ or CFZ respectively. LDH is associated with glucose metabolic pathways and modulated by TNF in PBMCs (113). These sets of data demonstrate that when 100 nM of BTZ is compared to 250 nM of CFZ, HPMECs display comparable cytotoxicity. However, ER stress and apoptotic signals appear to remain elevated in BTZ-treated conditions. We include in discussion the potential implications of this. Briefly, the half-life and biodistribution of BTZ are likely to contribute to enhanced toxicity of this drug *in vivo*. Our TNF data affirms synergy with BTZ or CFZ in HPMECs for different stress responses including apoptotic signal (**Figure 5C**) or metabolic effects (**Figure 5D**).

Our data identify three distinct patterns of cell stress response to proteasomal inhibitors-susceptibility in multiple myeloma cells, conditional susceptibility in microvascular endothelial cells and resistance in PBMCs. MM cells are highly susceptible to both reversible and irreversible proteasomal inhibition. In these cells, death is a CASP-dependent process. PBMCs resist cytotoxic effects of proteasomal inhibition at the applied dose. Endothelial cells are selectively sensitized to BTZ-induced death when compared to CFZ at same dose. In these cells, non-inflammatory cell death occurs due to buildup of intracellular stress uncoupled from extrinsic cues. The cytotoxicity is marked by increase of monolayer permeability, cell membrane permeability, proteotoxic stress, mitochondrial and endoplasmic reticular stress, as well as induction of apoptotic pathways. This condition synergizes with TNF present in the environment. CFZ also enhance its cytotoxic effects in HPMECs where TNF is present in the environment. When BTZ is compared to 2.5-fold higher dose of CFZ, cytotoxicity is comparable between two groups. However, stress markers (appearance of CHOP, cleaved product of PARP) continue to appear elevated. Physiologic levels of TNF continue to alter cellular response to proteasome inhibitors in this 1:2.5 dose ration of BTZ: CFZ. Together, this reflects a complex multi-cellular effect in the microvasculature when proteasome inhibitors are present and interplay of TNF with the endothelium. Our work further underscores the importance of considering half-life, clearance, and distribution when interpreting proteasome inhibitor effects on vascular cells.

## 4 Discussion

Peak plasma concentrations of CFZ or BTZ detected in patients are higher than 100 nM as discussed in results. Although cytotoxic effects were comparable when we studied the drugs scaled to clinically relevant concentrations, critical pharmacokinetic differences need to be considered. BTZ exhibits a long terminal half-life (~40-193 h, at 1.3 mg/m²) and wide tissue distribution, whereas CFZ has a very short half-life (<0.5 h, at 15-27 mg/m²) and rapid systemic clearance without accumulation. Additionally, BTZ’s volume distribution is~ 498–1884 l/m^2^ in comparison to a lower volume distribution for CFZ (~15–18 l/m^2^) (https://www.accessdata.fda.gov/drugsatfda_docs/label/2017/205004s000lbl.pdf and https://www.accessdata.fda.gov/drugsatfda_docs/label/2012/202714lbl.pdf). Consequently, CFZ achieves high transient plasma concentrations but is rapidly eliminated and less spread systemically, which will limit endothelial exposure. BTZ persists in circulation and peripheral tissues, increasing the potential for cumulative off-target toxicity. We postulate that this will result in BTZ exhibiting greater systemic toxicity *in vivo*, even though acute *in vitro* endothelial effects may appear similar. Our work is limited by the length of times assessed; we did not assess cytotoxicity beyond 24 h. We do not rule out elevated cytotoxicity of BTZ *in vitro* once extended time points are studied, given the appearance of elevated cleaved PARP in BTZ-treated cell lysates (**Figure 5C**). Irrespective of dose, we highlight that reversible or irreversible proteasomal inhibitors elicit multiple intracellular stress responses. Our work does not discern whether ER and mitochondrial stress pathways are parallel, sequential or synergistic to each other. CHOP-driven mitochondrial stress is a possibility that would implicate ER stress upstream of mitochondrial stress (114). CHOP, a developmentally regulated nuclear protein, triggers mitochondrial stress and apoptosis and can also collaborate with death receptors (DR-5, TRAIL) to activate CASP8 (115). Another possibility is that BTZ directly affects mitochondrial proteases. A previous study demonstrated that BTZ, but not CFZ, binds to and inhibits LonP1, a mitochondrial protease (116). LonP1 inhibition significantly activates the UPR and induces ER stress (117). There are some indications that additional mitochondrial proteases may be targeted by BTZ. A previous study in human neurons demonstrated that BTZ binds to mitochondrial proteases cathepsin (Cat) G, CatA, chymase, dipeptidyl peptidase II, and high-temperature requirement protein A2 (HtrA2)/Omi stress-regulated Endo protease. These findings argue for BTZ’s polypharmacologic effect and its ability to bind to multiple proteases. The authors conclude that HtrA2/Omi inhibition induces apoptosis in drug-treated cells (11). In a contrasting study, authors demonstrated that BTZ does not target HtrA2/Omi (118). There are more than 20 mitochondrial proteases, including HtrA2/Omi in the intermembrane space and LonP1 in the mitochondrial matrix, which are involved in stress responses at mitochondrial-associated membranes (MAMs). How proteasomal inhibition targets mitochondrial proteases in vascular/microvascular cells or other cell types require future-focused analyses.

Exogenous TNF enhances BTZ-mediated cytotoxicity on HPMECs (**Figures 3D, E**); whereas for CFZ, soluble factors from MM1.s or PBMCs are necessary along with TNF to exert cytotoxicity (**Figures 4D, E**). Physiologically relevant dose of TNF appears to elevate cleaved PARP signal and significantly alter cell-associated LDH levels suggesting multi-faceted contributions from this cytokine on microvascular cells. Given the non-inflammatory effect of CFZ or BTZ on MM1.s cells and PBMCs (**Figure 3**), we hypothesize that components in the supernatants are not inflammatory cytokines or chemokines. An alternative possibility is release of extracellular vesicle (EV)s by these cells. MM1.s cells produce EVs in the tumor microenvironment and CFZ or BTZ can both enhance EV production (119–121). Heightened EV production in the bone marrow disposes MM patients to poor prognosis and immune dysfunction (122–124). Under stress conditions, PBMCs also can produce EVs (125). Organ injuries are known to occur via EVs synergy with inflammatory cytokines (including TNF) to mediate cell death including that of endothelial cells (126, 127). This is currently an underexplored area, and we cannot predict whether CFZ or BTZ will be a better inducer of EVs. It is curious that BTZ-treated HPMECs do not display additional impact of supernatants+TNF (**Figure 4**). An explanation can be technical constraints. We detect cytotoxicity by measuring increased permeability of SYTOX Green fluorescent dye. BTZ alone kills ~50% of HPMECs (as determined in **Figure 1**). When exogenous TNF is added to BTZ, the remaining ~50% alive cells undergo cell membrane permeability at kinetics comparable to BTZ only conditions. It is likely that HPMECs exposed to (TNF+supernatant from cancer or immune cells)+BTZ (**Figure 4**) die faster due to presence of EVs or other materials in the supernatant. SYTOX Green incorporated cells are fragmented and cleared from the wells before detection. This is consistent with previous studies where SYTOX Green positivity decreases as cell death reaches 100% (51, 61). Accordingly, STS-treated HPMECs are all killed (**Supplementary Figures 1C, D**) and show less SYTOX Green+ cells. Overall, we clearly establish that proteasomal inhibition is cytotoxic to microvascular endothelial cells and TNF augments effects of both drugs studied. This likely plays out in Bortezomib-Induced Peripheral Neuropathy or CFZ-mediated adverse cardiovascular events noted in patients with (128–130). TNF is implicated in severity of BTZ-Induced Peripheral Neuropathy in patients (131). TNF is also known to associate with increased cardiovascular risk (132). It is important to reemphasize here that there is heterogeneity of TNF (89, 92, 93), CFZ or BTZ levels (52–54) in MM patients. This may explain why anti-TNF regimens, while showing promise in preclinical trials, are not mainstream clinical options yet (133). Sledge-hammer anti-inflammatory steroids remain cornerstone of supportive care along with proteasomal inhibitors (134). We predict that anti-TNF treatment may particularly benefit a subset of patients and not entirety of patient population. We do not resolve what cells produce TNF in the microvasculature. Neutrophils must be studied in future in this regard. These are the most abundant immune cell type in blood and undergo inflammatory cell death during stress (135). Neutropenia is a well-known clinical adverse effect of BTZ (136). MM also often associates with chronic infections (137). Neutropenia or infection can cause release of TNF in the bloodstream. We did not study direct cell-cell communication as would be expected to occur in vessels and microvessels, Future experimentation where MECs are co-cultured with MM cells, PBMCs or neutrophils will be necessary to establish the full breadth of effect of CFZ or BTZ in the microvessels.

How microvascular events contribute to immunity, homeostasis and organ injury is an emerging concept (138–141). Neurotoxic and neurodegenerative conditions are prominent examples of injury where vascular/microvascular damage contributes (138, 142–144). This includes peripheral neuropathy (145, 146), a prominent known adverse effects of intravenous BTZ administration (13, 147–149). This is reported in ~36-75% patients as primary adverse complication for BTZ (33, 34, 147, 150, 151). CFZ associates with lesser incidences of neuropathy (148, 152). Cardiovascular adverse effects and renal toxicities are more prevalent for CFZ (12, 152, 153). Even though we do not explore sequalae of events, our observations lay foundation for tentative tissue injury model driven by CFZ or BTZ in combination with TNF (**Supplementary Figures 4A, B**). As an example demonstration of tissue injury, we illustrate how PIs may cause peripheral blood-nerve-barrier damage (**Supplementary Figure 4B**). Microvessels of the endoneurium sustain blood flow to the nerves (154). We hypothesize that microvascular damage (which by itself does not activate the endothelium or make them inflammatory) drives blood leukocyte infiltration causing neuroinflammation and degeneration. TNF amplifies this injury. Consistently, clinical observations and rodent models implicate TNF in BTZ-mediated neuropathies (32–35). Patients benefit by combination treatment of PIs with immunosuppressive agents like dexamethasone and cyclophosphamide (155, 156). While we do not address all bystander effects individually, the identified mechanisms may contribute to other clinical adverse effects (gastrointestinal, renal, cardiovascular, pulmonary complications and thrombocytopenia) (157).

Our standalone observations are relevant for understanding microvascular endothelial cell functions beyond chemotherapy. Proteasomal inhibition is a natural component of aging and associates with age-related neurodegenerative diseases (158–160). Inflammatory cytokines, including TNF, strongly associate with disease development (161, 162). Primary studies identifying association of proteasomal inhibition with aging focus on neurons. There is very limited understanding of how natural proteasomal inhibition dictates microvasculature environment. Our work puts forward the idea that BTZ or CFZ treatment of cells could be used to study the natural process (163) in microvasculature. Whether CFZ or BTZ mimic the natural process remains to be investigated. In the aging brain as regular proteasomal activities decrease, inflammatory immunoproteasome activity increases (164). This argues for the potential of approved proteasomal inhibitors as anti-neurodegenerative drugs, and we provide insights into potential bystander effect for those clinical applications as well. Overall, we bring to light how circulating multiple myeloma cells (165, 166) and PBMCs (11, 167) may communicate with endothelium during proteasomal inhibition. For the clinical context, we also want to highlight the socio-economic aspects of multiple myeloma management (168). BTZ is more affordable than CFZ, with generic versions available. Policymakers should consider updated data to create cost-effective strategies without compromising clinical outcomes or increasing adverse effects. Alternative, second-generation reversible proteasomal inhibitors, such as Ixazomib, prescribed as oral medication, should be investigated for broader clinical use and associated bystander effect (169). In broader biology, this work explains how proteasomal stress and degradation pathways contribute to disease beyond cancer treatment.

## Data Availability

All data and any code are available through requests made to lead contact Dr. Pratyusha Mandal (pratyusha.mandal@lehman.cuny.edu).

## Supplementary material

Supplementary Figure 1 Distinct permeability but similar viability responses to proteasome inhibitors across microvascular endothelial cells.

Supplementary Figure 2 Bortezomib induces more ROS and earlier proteotoxic stress than carfilzomib.

Supplementary Figure 3 Proteasome inhibitor sensitizes multiple myeloma cells and triggers caspase-dependent death.

Supplementary Figure 4 Model for chemotherapeutic proteasomal inhibition induced cytotoxicity **(A)** and tissue injury.
