# Supplementary material for: Proteasomal inhibition compromises microvascular integrity via distinct effects on non-immune endothelial cells and immune cells
Source: Front Immunol. 2026 May 19;17:1759368. doi: 10.3389/fimmu.2026.1759368 (PMC13238444; doi:10.3389/fimmu.2026.1759368)
Supplement: Supplementary Figure 1 — Distinct permeability but similar viability responses to proteasome inhibitors across microvascular endothelial cells. [file DataSheet1.docx]

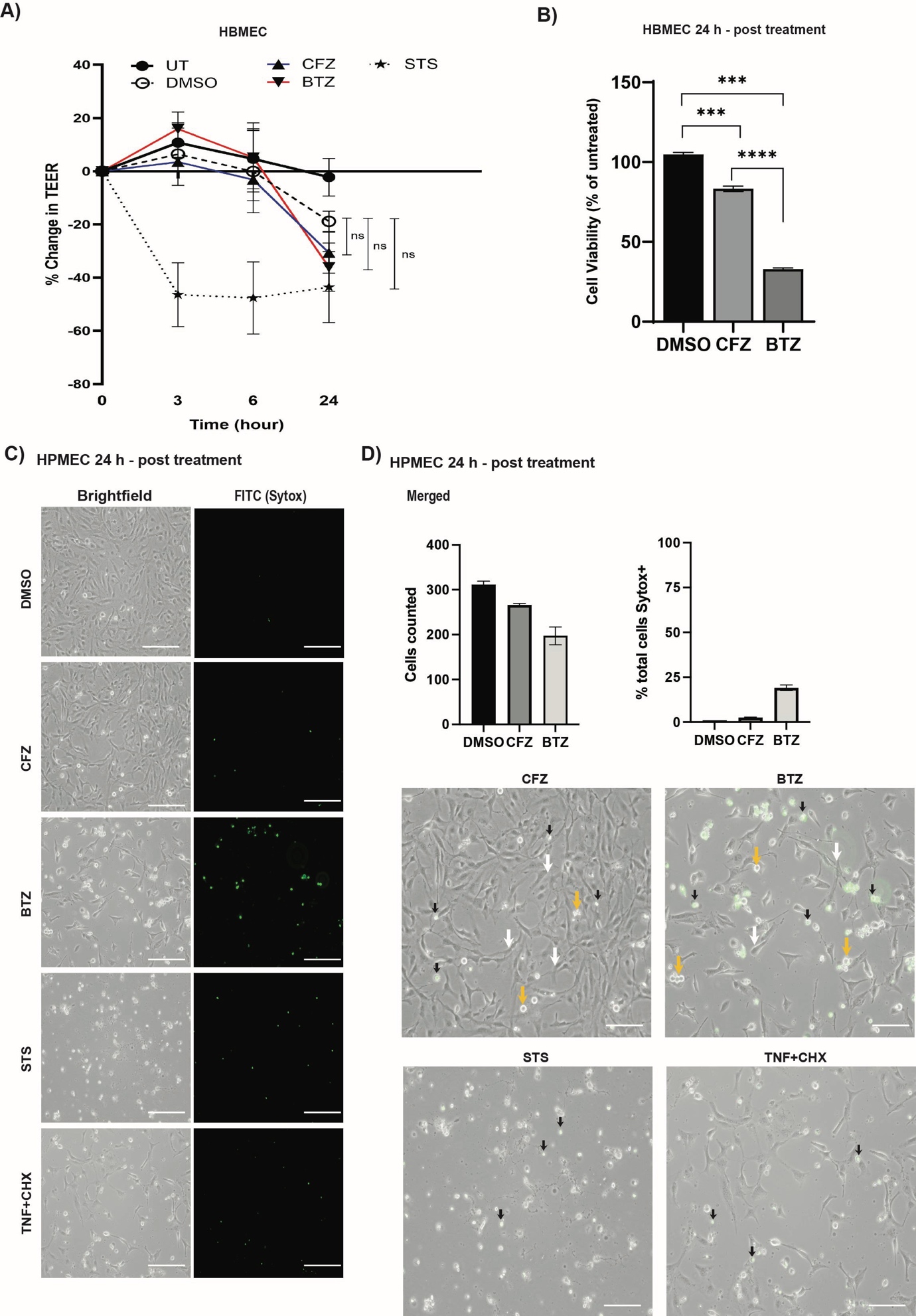


**Supplemental Figure. 1. Distinct permeability but similar viability responses to proteasome inhibitors across microvascular endothelial cells. (A)** Endothelial barrier integrity was assessed in human brain microvascular endothelial cells (HBMECs) by transendothelial electrical resistance (TEER). HBMEC monolayers treated were left untreated (UT) or treated with DMSO, CFZ (100 nM), BTZ (100 nM), or STS (1 μM) at indicated times. Data represent percent change in TEER relative to baseline (error bars indicate mean ± SEM, N=3 independent experiments). Statistical analysis using two-way ANOVA with multiple comparisons showed no significant differences between DMSO and BTZ or CFZ at 24 h post treatment. ns is non-significant. **(B)** **Cell viability was assessed in HBMEC by quantification of ATP levels** 24 h post treatment with DMSO, CFZ (100 nM) or BTZ (100 nM). Data represent percentage of viable cells relative to untreated (error bars represent mean ± SEM, N= 6-12 wells from one experiment). Statistical significance was evaluated using unpaired, non-parametric Mann-Whitney test. CFZ vs. DMSO is ***p= 0.0001, BTZ vs. DMSO is ***p= 0.0001, BTZ vs CFZ is ****p< 0.0001. **(C)** Morphological changes and membraned compromise was assessed using brightfield microscopy and SYTOX Green fluorescence signal (FITC) respectively. Representative (from N=3 independent experiments) brightfield images and SYTOX Green (FITC) of HPMECs treated for 24 h with DMSO, CFZ (100 nM), BTZ (100 nM), STS (1 μM), TNF (25 ng/ml) plus CHX (1 μM). Scale bar = 170 μm. **(D)** Total cells (alive + dead) visible in brightfield images were counted using ImageJ Cell Counter Plugin (upper left panel). To detect SYTOX+ dying cells, FITC (SYTOX Green)+ cells were counted from merged brightfield and fluorescent images (upper right panel). Error bars indicate mean ± SEM, N= 3 independent images. There is no indicated significance as none of the shown conditions are significantly different using unpaired, non-parametric Mann-Whitney test. Microscopy images are magnified merged (brightfield+FITC) images of CFZ, BTZ, STS, TNF+CHX treatment conditions shown in Supplementary Figure 3C . White arrows point to representative alive cells, yellow arrow points to representative dying cells and black arrow points to representative SYTOX Green+ cells.


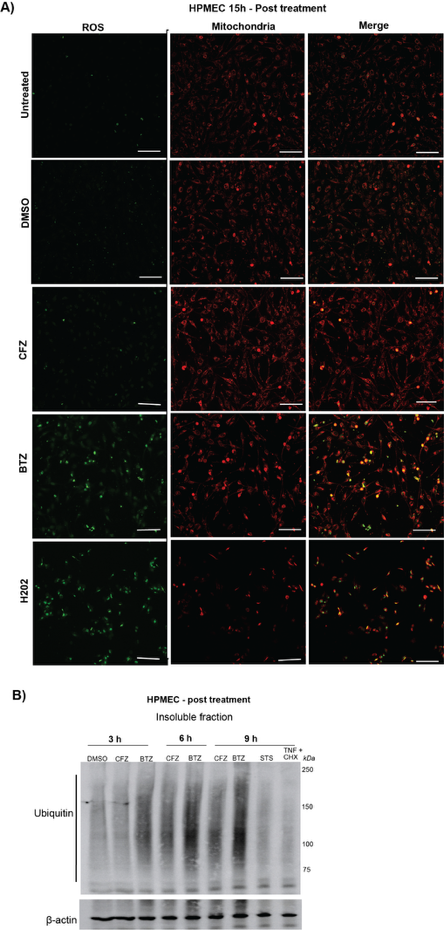


**Supplemental Figure. 2. BTZ induces enhanced appearance of ROS and earlier proteotoxic stress when compared to CFZ. (A)** Qualitative assessment of ROS and mitochondrial localization was performed in HPMECs. Cells treated with CFZ (100 nM), BTZ (100 nM), or controls (DMSO, H₂O₂ 500 μM) were stained with CellROX Green and MitoTracker Red. Representative (from N=3 independent experiments) images show ROS (green), mitochondria (red), and merged signals indicating ROS localization within mitochondria. Scale bar=170 μm. **(B)** Immunoblot of HPMEC cells shows expression of total ubiquitin indicating proteotoxic stress in insoluble protein fractions collected at 3, 6, and 9 h post treatment. Smears indicate accumulation of ubiquitinated proteins (75kDa to 250kDa) with β-actin (45 kDa) as loading control (N=1 experiment).

**
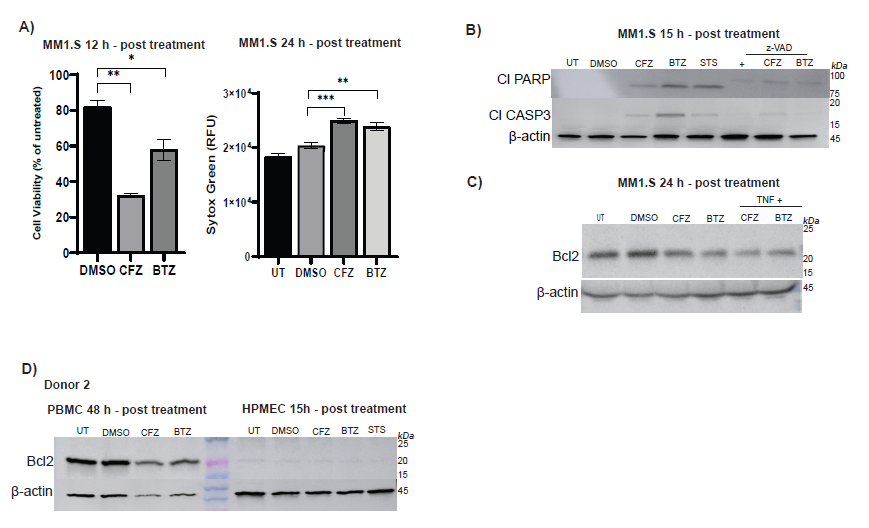
**

**Supplemental Figure. 3. Proteasome inhibitors sensitize MM cells and triggers CASP-dependent death. (A**) Cell viability was assessed based on loss of ATP for MM1.S after 15 h (left panel) and 24 h (right panel) of treatment with CFZ (100 nM), BTZ (100 nM) or DMSO (for 15 h) along with untreated (UT) for 24 h. Data are presented as viability percentage relative to untreated cells (error bars represent mean ± SEM, N=6-12 wells from one experiment) Statistical comparison performed using unpaired, non-parametric Mann-Whitney test. For 15 h (left panel) CFZ vs. DMSO is **p< 0.01, BTZ vs. DMSO is *p=0.01. For 24 h (right panel), CFZ vs. DMSO is ***p=0.0002, BTZ vs. DMSO is **p= 0.001. **(B)** Representative (from N=2 independent experiments) immunoblot analyses of MM1.S cells left untreated (UT) or after 15 h of treatment with DMSO, CFZ (100 nM), BTZ (100nM), STS (1 μM), zVAD (20–25 μM) alone or in combination with CFZ (100nM) or BTZ (100 nM) show expression of apoptosis markers: Cl-CASP3 (17, 19 kDa), Cl-PARP (89 kDa) with β-actin (45 kDa) as loading control**. (C)** Representative (from N=1 experiment) immunoblot analyses show MM1.S cells left untreated (UT) or after 15 h of treatment 24h with DMSO, CFZ (100nM), BTZ (100nM), TNF (25 ng/ml) plus CFZ (100 nM), TNF plus BTZ (100 nM), showing expression of BCL2 (26kDa) with β-actin (45 kDa) as loading control. **(D)** Representative (from N=2 experiments) immunoblot analyses show PBMCs (Donor 2) (left side of the ladder) left untreated (UT) or after 48 h of treatment with DMSO, CFZ (100nM), BTZ (100nM) and HPMEC (right side of ladder) left untreated (UT) or after 15 h of treatment with DMSO, CFZ (100nM), BTZ (100nM) and STS (1 μM) showing expression of BCL2 (26kDa) with β-actin (45 kDa) as loading control.

**A)**

**
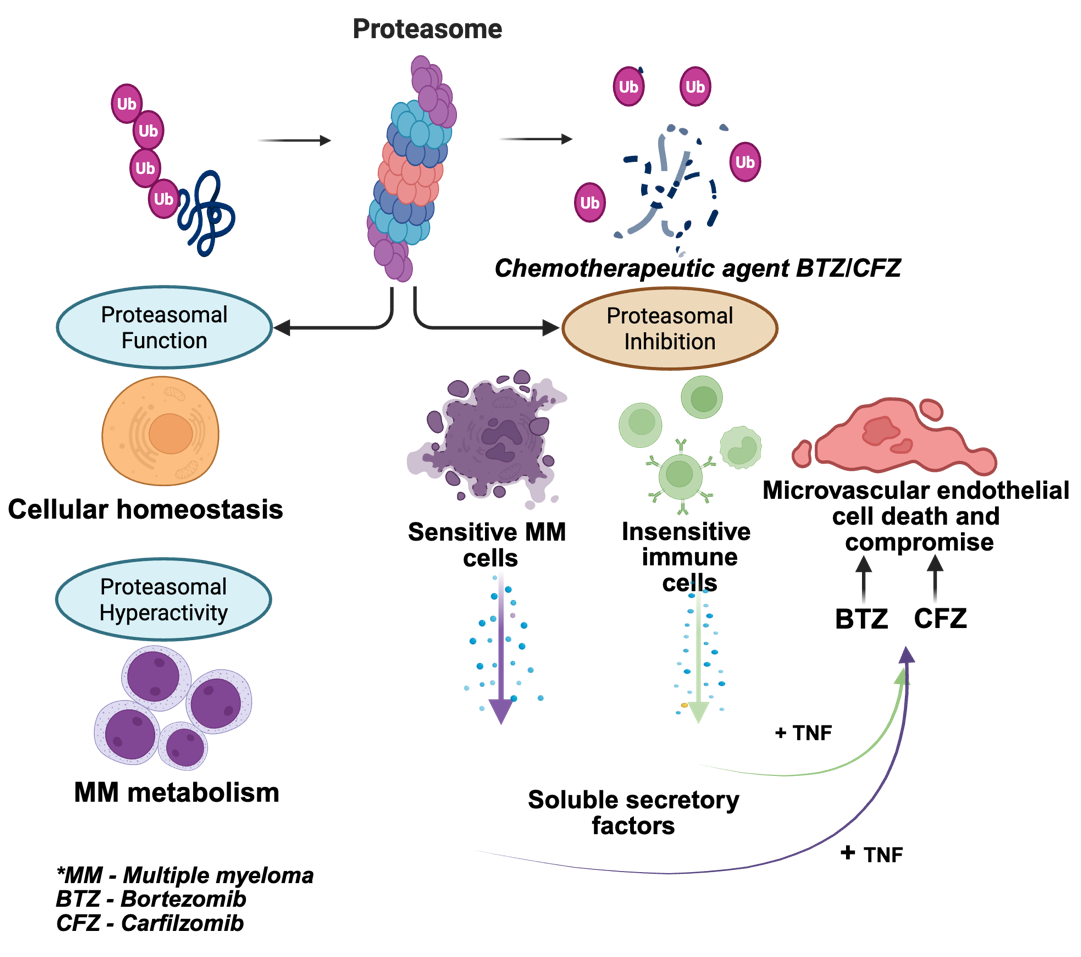
**

**B)**

**
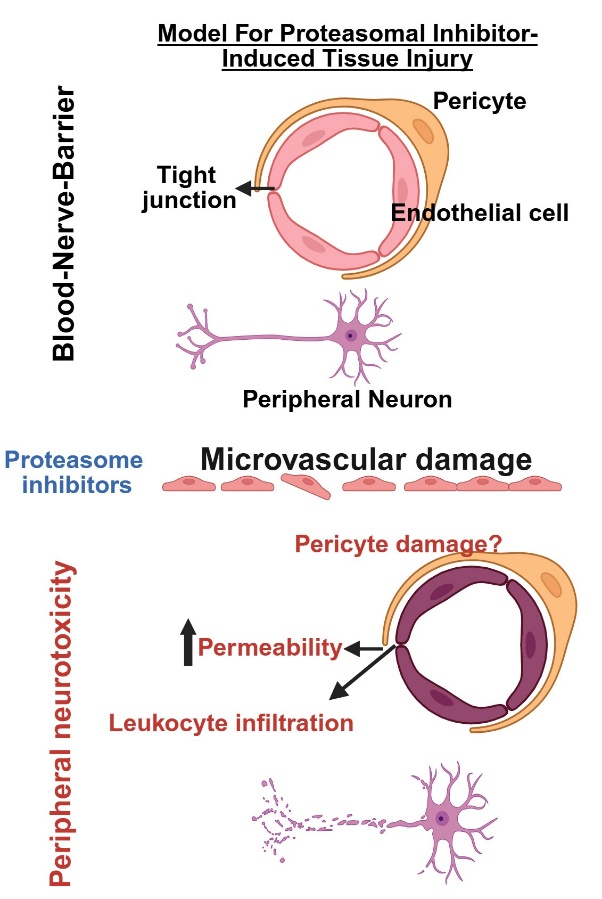
**

**Supplemental Figure.** **4. Model for chemotherapeutic proteasomal inhibition-induced cytotoxicity (A) and tissue injury (B). A**) Proteasome inhibitors BTZ and CFZ are cytotoxic for multiple myeloma (MM) and microvascular endothelial cells. Inflammatory cytokine TNF synergizes with factors derived from MM cells and immune cells proteasome inhibitor compounds to enhance microvascular cytotoxicity. **B**) Model illustration shows how proteasomal inhibitors can potentially drive drug-associated neuropathology via microvascular damage even though the current data did not directly address this. Microvascular damage increases permeability of the endothelium. This breaches the blood-nerve-barrier causing leukocyte infiltration and inflammatory neurodegeneration. Effects on associated cells including pericytes deserve future attention. Figure created using BioRender.
